# A Cross‐Linked Flexible Metaferroelectrolyte Regulated by 2D/2D Perovskite Heterostructures for High‐Performance Compact Solid‐State Sodium Batteries

**DOI:** 10.1002/advs.202416662

**Published:** 2025-06-23

**Authors:** Yanan Huang, Zhuo Yang, Weicheng Zhou, Liequan Liu, Chuanbao Tu, Mingyang Tang, Haijiao Xie, Yu Lu, Xu Yan, Zhihao Ding, Xiaolong Li, Tiannan Yang, Alexander S. Sigov, Wei Huang, Lijun Gao, Cheng Huang

**Affiliations:** ^1^ Volta and DiPole Materials Labs, College of Energy, Soochow Institute for Energy and Materials InnovationS (SIEMIS), Soochow Innovation Consortium for Intelligent Fibers and Wearable Technologies, Key Laboratory of Advanced Carbon Materials and Wearable Energy Technologies of Jiangsu Province, Key Laboratory of Core Technology of High Specific Energy Battery and Key Materials for Petroleum and Chemical Industry Soochow University 688 Moye Road Suzhou 215006 P. R. China; ^2^ International Joint Metacenter for Advanced Photonics and Electronics, Physics and Energy Department, School of Optical and Electronic Information Suzhou City University 1188 Wuzhong District Suzhou 215006 P. R. China; ^3^ High Density Materials Technology Center for Flexible Hybrid Electronics, Innovation Center for MIIT China Prosperity Green Industry Foundation & Industry Development Research Institute, Xi'an Jiaotong University‐Suzhou Institute of Electronic Functional Materials Technology Suzhou Industrial Technology Research Institute Suzhou 215151 P. R. China; ^4^ Interdisciplinary Research Center, School of Mechanical Engineering Shanghai Jiao Tong University 800 Dongchuan Road Shanghai 200240 P. R. China; ^5^ School of Flexible Electronics & State Key Laboratory of Optoelectronic Materials and Technologies Sun Yat‐sen University 66 Gongchang Road, Guangming District Shenzhen 518107 P. R. China; ^6^ Nanoelectronics Department MIREA‐Russian Technological University 78 Vernadsky Ave. Moscow 119454 Russia; ^7^ Shanghai Synchrotron Radiation Facility, Shanghai Advanced Research Institute, Shanghai Institute of Applied Physics Chinese Academy of Sciences Shanghai 201204 P. R. China; ^8^ Pacific Northwest National Laboratory Richland WA 99352 USA; ^9^ Institute of Advanced Materials and Institute of Membrane Science and Technology, Jiangsu National Synergistic Innovation Center for Advanced Materials, State Key Laboratory of Flexible Electronics Suzhou Laboratory and Nanjing Tech University Nanjing 211816 P. R. China

**Keywords:** 2D ferroelectric heterostructures, Conformal battery interface, Fast‐ion conductor, Ferroelectric polymer, Metaferroelectrolyte, Solid‐state sodium battery (SSB) energy storage, Strong ferro‐electrochemical coupling

## Abstract

To address the issues of limited ionic conductivity and poor interface stability at room and low temperatures in solid‐state electrolytes, a robust intrinsic ferroelectrolyte or nanoferroelectrolyte strategy for engineering solid‐state flexible ferroelectric composite electrolytes utilizing strongly coupled intrinsic ion conducting 2D/2D sodium‐rich anti‐perovskite (NaRAP)/ferroelectric perovskite heterostructures is introduced. Herein, highly scalable PVDF‐based metaferroelectrolytes with Na_2.99_Ba_0.005_OCl/Ca_2_Na_2_Nb_5_O_16_^−^ (CNNO^−^) nanosheets into a ferroelectric poly(vinylidene fluoride‐co‐hexafluoropropylene) (PVDF‐HFP) matrix, through an in situ cross‐linking and spontaneous bridging method, for compact solid‐state sodium batteries (SSBs), are reported. Benefiting from unique well‐dispersed 3D ferroelectric coupled network and the Na_2.99_Ba_0.005_OCl/CNNO^−^‐induced PVDF‐HFP ferroelectric β phase, the Na^+^ flux is regulated, thereby inhibiting Na dendrite growth at the interface. Notably, the optimized PH‐5% NC metaferroelectrolyte exhibits rapid ion transport (1.11 × 10^−4^ S cm^−1^ at 25 °C), a wide electrochemical window (> 4.8V), superior conformal mechanical compatibility, improved flexibility, good elasticity and flame retardancy. The solid‐state Na_3_V_2_(PO_4_)_3_/PH‐5% NC/Na batteries present a stable cycling performance (remaining 56.4 mAh g^−1^ after 500 cycles at 1 C) even at 0 °C, potential for cost‐effective, safe, stable and compact SSB energy storage over 600 Wh L^−1^, vastly surpassing 365 Wh L^−1^ of the current commercial sodium‐ion liquid‐electrolyte batteries.

## Graphical abstract

The flexible solid‐state metaferroelectrolyte is fabricated through an in situ cross‐linking and spontaneous bridging method by introducing strongly ferroelectric‐coupled intrinsic ion conducting Na_2.99_Ba_0.005_OCl/CNNO^‐^ nanosheets into PVDF‐HFP matrix. The unique 3D ferroelectric‐coupled network and Na_2.99_Ba_0.005_OCl/CNNO^‐^‐induced ferroelectric PVDF‐HFP β phase effectively enhance ion transport, inhibit sodium dendrite growth, and exhibit good conformal mechanical compatibility, fireproofing properties, low‐temperature performance and energy‐dense srorage for compact energy storage.

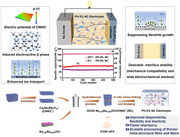

## 1 Introduction

The burgeoning demand for large‐scale energy storage has propelled the energy industry to innovate and pursue novel storage technologies that are not only cost‐effective, but also ensured the safety.^[^ ^1^, ^2^ ^]^ Solid‐state alkali metal batteries have revolutionized the field of energy storage, representing a promising alternative to traditional liquid‐electrolyte batteries, owing to their unparalleled safety and higher energy density. Solid‐state sodium batteries (SSBs), utilizing non‐flammable composite electrolytes, are becoming the most prospective next‐generation energy storage devices.^[^ ^3^, ^4^, ^5^ ^]^ However, SSBs still suffer from the sluggish ionic kinetics, fatigue behaviors and poor interface stability of solid electrolytes, especially at low temperatures. Note that the severe low‐temperature environment would further deteriorate the electrolyte kinetics. In this scenario, the key point to broaden the application prospect of SSBs also lies on exploring the desirable electrolyte materials that possess fast ionic kinetics and compatible electrode‐electrolyte interfaces even in severe environments. Solid‐state electrolytes are primarily categorized into inorganic^[^ ^6^, ^7^ ^]^ and polymer solid electrolytes. Among the inorganic solid electrolytes, sulfides exhibit exceptional ionic conductivity.^[^ ^8^, ^9^ ^]^ However, current sulfide electrolytes may generate hydrogen sulfide upon exposure to air, triggering detrimental side reactions with Na anodes.^[^ ^10^, ^11^ ^]^ These reactions can result in the decomposition of sulfides into poor ionic conductors such as Na_3_P and Na_2_S, which exhibit poor compatibility with Na anodes.^[^ ^12^ ^]^ In comparison, oxide solid electrolytes exhibit remarkable safety features and an extensive electrochemical window,^[^ ^13^, ^14^ ^]^ but the conductivity of sodium ions is an order of magnitude lower than that of sulfides.^[^ ^15^ ^]^ Oxyhalide solid‐state electrolytes are a breakthrough in energy storage incorporated with the advantages of both halide and oxide electrolytes. Oxyhalide solid‐state electrolytes based on sodium‐rich anti‐perovskites (NaRAPs),^[^ ^16^, ^17^ ^]^ a class of sodium superionic conductors,^[^ ^18^, ^19^ ^]^ have garnered immense interest in the field of solid‐state electrolyte research owing to their high theoretical ionic conductivity, negligible electronic conductivity, wide electrochemical window, robust mechanical properties, and cost‐effective (only for NaOA). Their distinguishing feature is their anti‐perovskite structures.^[^ ^20^, ^21^ ^]^ The exploration of NaRAP (Na_3_BA, where A = Cl, Br, etc.)  dates back to the 1990s, when Müller et al. unveiled an anti‐perovskite structure exhibiting Na^+^ conductivity.^[^ ^22^ ^]^ The synthesized Na_3_OBr served as a high‐temperature Na^+^ conductor, with an ionic conductivity ranging from 10^−5^ to 10^−4^ S cm^−1^ at a high temperature of 230 °C. NaRAP generally possesses a layered anti‐perovskite crystalline or glassy amorphous ferroelectric structure similar to traditional perovskites but with an inverted electronic lattice arrangement, where Na^+^ occupies octahedral vertices and divalent anions (typically O or S) are located at octahedral centers. The monovalent anions at the dodecahedral center (A site) can encompass halogens (F, Cl, Br, and I), polyanions (e.g., BH_4_^−^), or a mixture of these.^[^ ^23^ ^]^ Notably, NaRAP electrolytes can host a substantial quantity of Na^+^ within their lattice and enable the easy movement of Na^+^ by introducing crystal defects, such as Na^+^ and Cl^−^ vacancies. Typical divalent cation dopants include Mg^2+^, Ca^2+^, Sr^2+^,^[^ ^16^ ^]^ Ba^2+^,^[^ ^24^ ^]^ and trivalent Al^3+^. Established anti‐perovskite electrolytes, including Li_3_OCl,^[^ ^25^, ^26^ ^]^ Li_3_OBr,^[^ ^27^ ^]^ Na_3_OBr,^[^ ^28^ ^]^ and Na_4_OI_2_,^[^ ^29^ ^]^ exhibit good electrochemical compatibility with Li or Na anodes, but their practical ionic conductivities still remain low. Their high brittleness (not easy to process and poor interface contact) and moisture instability (and then poor cycle life) impede their applications for sustainable all solid‐state batteries. Consequently, the design and fabrication of novel anti‐perovskite electrolytes through structural modifications is needed. Thus, enhancing the ionic conductivity, structural stability, and electrode‐electrolyte interface stability of solid‐state electrolytes at room temperature and below has always been a critical issue in solid‐state batteries.^[^ ^30^, ^31^ ^]^

While a gap still exists between the requirements for solid‐state batteries applications and the current conductivity and interface stability of NaRAP, its intrinsic structural attributes are conducive to designing and synthesizing NaRAP‐type electrolytes with desirable ionic conductivity and interface stability. Inspired by the exploration and utilization of electroactive 2D layered heterostructures, this challenge might be resolved through structural engineering and composition optimization. Two‐dimensional layered heterostructures including van der Waals or ionic or charge‐type layered materials offer superior versatility.^[^ ^32^ ^]^ Van der Waals layered materials composed of electrically neutral 2D units can exhibit novel physical properties owing to the differences in interlayer stacking patterns, rotational angles, and other factors. By contrast, ionic or charge‐type layered materials, such as LaOFeAs, comprise interleaved layers of charged [La_2_O_2_]^2^⁺ and [Fe_2_As_2_]^2^⁻ units that enable functional‐oriented design and property modulation.^[^ ^33^, ^34^ ^]^ Owing to the diversity of ionic layers, different interlayer electronic structure couplings and charge transfers are expected to give rise to novel physical properties and superior performances not present in van der Waals layered materials. Similarly, battery electrolyte systems with strong ferroelectric‐electrochemical coupling are being developed to enhance interfacial ion transport and optimize ferroelectric ions kinetics.

Ferroelectric materials,^[^ ^35^ ^]^ utilizing unique spontaneous dipole polarization and multi‐field coupling, offer tailored functionalities for advanced energy storage and conversion systems, especially lithium‐ion batteries.^[^ ^36^, ^37^ ^]^ Their ferroelectric effect and ferro‐electrochemical coupling are expected to enhance alkali‐ion kinetics significantly, probably through the “initiative” accelerators. Kang et al. synthesized porous ferroelectric ceramic Bi_4_Ti_3_O_12_ nanofibers to establish swift conductive pathways for Li^+^ in a PEO/LiTFSI system.^[^ ^38^ ^]^ This strategy led to a remarkable enhancement in lithium‐ion conductivity (6.25 × 10^−4^ S cm^−1^ at 50 °C). Huang et al. incorporated the ferroelectric PbZr_x_Ti_1−x_O_3_ (PZT) nanoparticles into poly(vinylidene fluoride).^[^ ^39^ ^]^ The high dielectric constant of PZT facilitates the uniform distribution of numerous dipoles. These dipoles attract and promote ion transport along the dipolar channels, significantly enhancing lithium salt dissociation into free Li^+^ and promoting efficient ion transport. Incorporating and coupling with ceramic ferroelectrics or dielectrics with ion conductors has enhanced Na^+^ mobility, yet the strong coupling in intrinsic ferroelectrolytes faces challenges due to the ionic insulation of ferroelectric fillers in composite structures. Utilizing 2D ferroelectric material and electrolyte nanosheets as building blocks, atomic‐layer engineering and nanoarchitectonics enable the construction of 2D layered perovskite heterostructures. These structures give rise to intrinsic 2D ferroelectrolytes and even metaferroelectrolytes, where novel 2D strongly coupled mechanisms emerge. Ca_2_Na_2_Nb_5_O_16_^−^ (CNNO^−^), as a typical layered‐perovskite ferroelectric material, possesses a large dielectric constant (ε_r_≈390).^[^ ^40^, ^41^ ^]^ Its highly polarized NbO_6_ octahedral units generate a built‐in electric field (EF) which might provide a uniform, rapid Na^+^ transport mechanism when strongly coupled with layered NaRAPs. Thus, 2D intrinsic ferroelectrolytes comprise interleaved layers of ferroelectric perovskite CNNO^−^ and sodium‐rich anti‐perovskite Na^+^RAP units. And these charged ionic units can be classified into three categories: the property‐determining layer (CNNO^−^ ferroelectric layer) and the carrier reservoir layer (Na^+^RAP ionic electrolyte layer) as well as strongly coupled interfaces. With the development of functional solid‐state electrolytes possessing the ability to regulate EF levels and ion fluxes, the perovskite heterostructures with strong ferro‐electrochemical coupling own the greater potential to be served as intrinsic ion conducting ferroelectrolytes for solid‐state batteries, while it has not been revealed experimentally up until now.

A robust intrinsic ferroelectrolyte or nanoferroelectrolyte strategy is introduced, utilizing strongly coupled intrinsic ion conducting 2D/2D NaRAPs/ferroelectric perovskite heterostructures to engineer solid‐state flexible ferroelectric composite electrolytes, addressing low ionic conductivity and poor interface stability at room and low temperatures. Herein, we report highly scalable and flexible solid‐state PVDF‐based metaferroelectrolytes (PH‐x% NC) with Na_2.99_Ba_0.005_OCl/CNNO^−^ nanosheets into a ferroelectric poly(vinylidene fluoride‐co‐hexafluoropropylene) (PVDF‐HFP) matrix, through an in situ cross‐linking and spontaneous bridging method, for compact SSB. The intrinsic ion conducting 2D/2D layered perovskite heterostructures were fabricated by layer‐by‐layer self‐assembly of coupled glassy Na_2.99_Ba_0.005_OCl ion conductors and ferroelectric CNNO^−^ nanosheets. The strong interface coupling enhanced intrinsic ionic conductivity and air stability of entire electrolytes material. PVDF‐HFP possesses a nature of robust tensile strength but weak toughness. Nevertheless, by chemical cross‐linking and subsequently spontaneous bridging, its flexibility and elasticity were significantly improved for better rough and resilient conformal interfacial contact. The optimized PH‐5% NC metaferroelectrolyte features a 3D interpenetrating ferroelectric network, providing an efficient permeation pathway for ions. The built‐in EF promotes free Na^+^ release for transport along the highly conductive ionic layers, realizing uniform deposition of Na. The ionic conductivity and sodium transference number of the prepared PH‐5% NC metaferroelectrolyte attained 1.11 × 10^−4^ S cm^−1^ and 0.71, respectively, at 25 °C. The assembled Na|PH‐5% NC|Na symmetric cells maintained superior cycle stability for more than 1 000 h at 25 °C. The Na_3_V_2_(PO_4_)_3_/PH‐5% NC/Na cell delivered stable cycling performance (56.4 mAh g^−1^ after 500 cycles at 1 C) at 0 °C. Our findings provide new insights into the strongly coupled system fundamentals and applications of PVDF‐based solid‐state metaferroelectrolytes for compact sodium battery energy storage over 600 Wh L^−1^.

## 2 Results and Discussion

### 2.1 Design and Fabrication of the Flexible Solid‐State Metaferroelectrolytes

**Figure 1:**
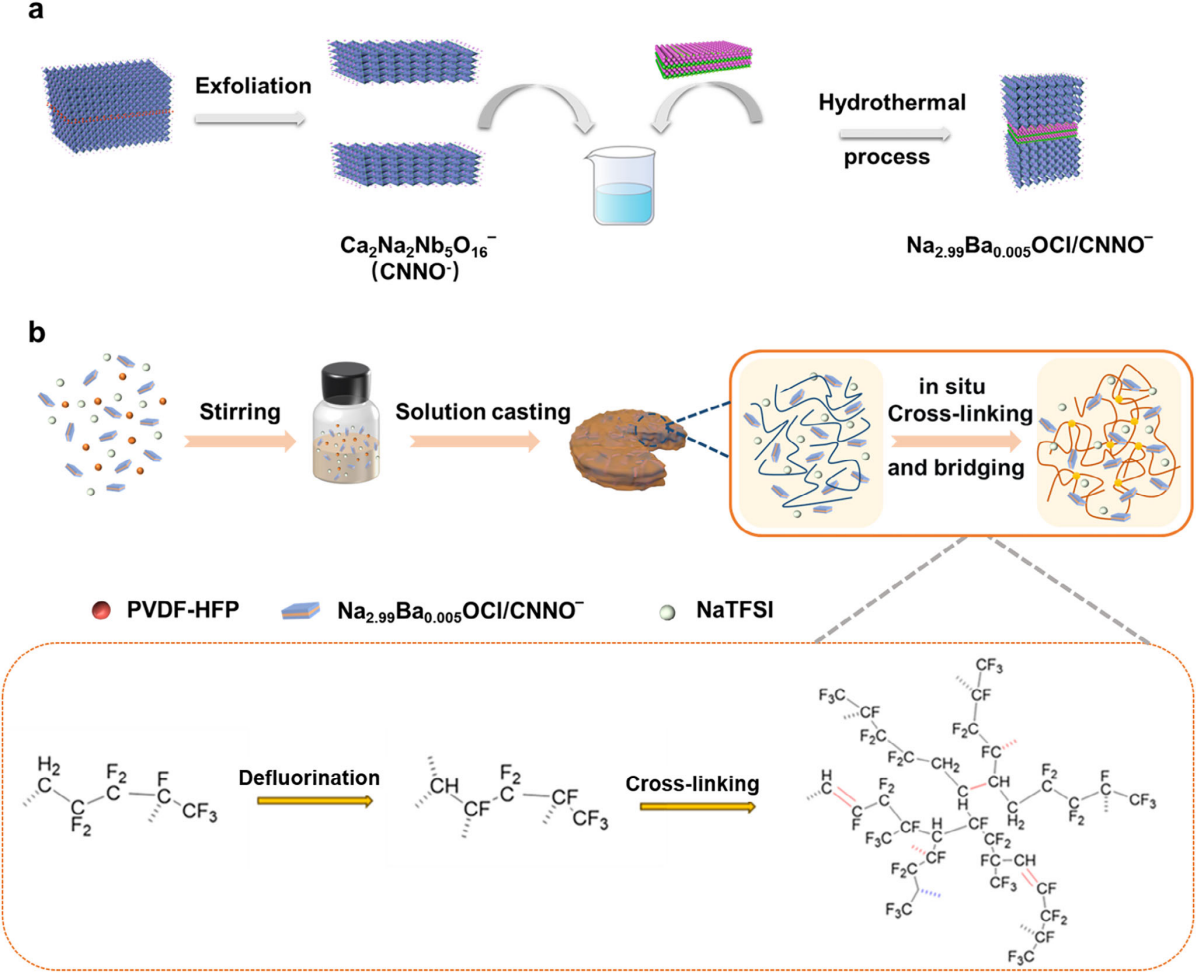
Schematic illustration of the metaferroelectrolyte design and fabrication. a) Strongly coupled intrinsic ion conducting 2D/2D sodium‐rich anti‐perovskite (NaRAP) / ferroelectric perovskite heterostructure ferroelectrolyte, Na_2.99_Ba_0.005_OCl/CNNO^‐^. b) in situ cross‐linked and spontaneously bridged solid‐state flexible metaferroelectrolyte, PH‐5% NC.

A facile, scalable approach to the synthesis of Na_2.99_Ba_0.005_OCl/CNNO^−^ heterostructures is schematically depicted in **Figure** **1**a. The KCa_2_Na_2_Nb_5_O_16_ powder obtained after high‐temperature calcination was exfoliated by the liquid‐phase method using interlayer cations, such as H^+^ and TBA^+^, to obtain Ca_2_Na_2_Nb_5_O_16_^−^ nanosheets. Na_2.99_Ba_0.005_OCl/CNNO^−^ was fabricated using a simple one‐pot hydrothermal method. To verify the air stability enhancement of Na_2.99_Ba_0.005_OCl/CNNO^−^, air exposure tests were performed (25 °C, 24h). The results showed significant deliquescence on the surface of Na_2.99_Ba_0.005_OCl, while the Na_2.99_Ba_0.005_OCl/CNNO^−^ powder maintained structural integrity in Figure S1 (Supporting Information). The comparison results confirm that the Na_2.99_Ba_0.005_OCl/CNNO^−^ heterostructure materials have better air stability. Figure 1b illustrates the preparation of the fireproof PH‐5% NC metaferroelectrolyte through in situ cross‐linking and spontaneous bridging of PVDF‐HFP chains, where Na_2.99_Ba_0.005_OCl served as the initiator and ionic conductor within Na_2.99_Ba_0.005_OCl/CNNO^−^. Interestingly, when fluorine and hydrogen atoms are attacked by nucleophilic reagents (such as the alkaline compound Na_2.99_Ba_0.005_OCl), defluorination and cross‐linking occur in the PVDF‐HFP chains. Consequently, the ferroelectric network was established by chemical cross‐linking of PVDF‐HFP chains, and good intrinsic elasticity of polymer electrolytes was achieved. The increase of internal elasticity can effectively adapt to the volume change of the active materials during the cycling, and enhance the lasting stability of the interface. The pre‐dehydrofluorination reaction during polymer synthesis enhanced the structural stability, slowing the degradation of the PVDF‐HFP polymers during the charge/discharge process. Digital photos of the samples are provided in Figure S2 (Supporting Information) for further details of cross‐linking with different Na_2.99_Ba_0.005_OCl/CNNO^−^ content, which revealed the thickness of PH‐5% NC electrolyte (≈53 µm). However, over‐cross‐linking prevented the polymer from forming a film. In contrast, PH‐5% N composite electrolyte of the same thickness was synthesized using a similar tape‐casting method without CNNO^−^.

### 2.2 Characterization of Na_2.99_Ba_0.005_OCl/CNNO^−^ Heterostructures and Metaferroelectrolytes

**Figure 2:**
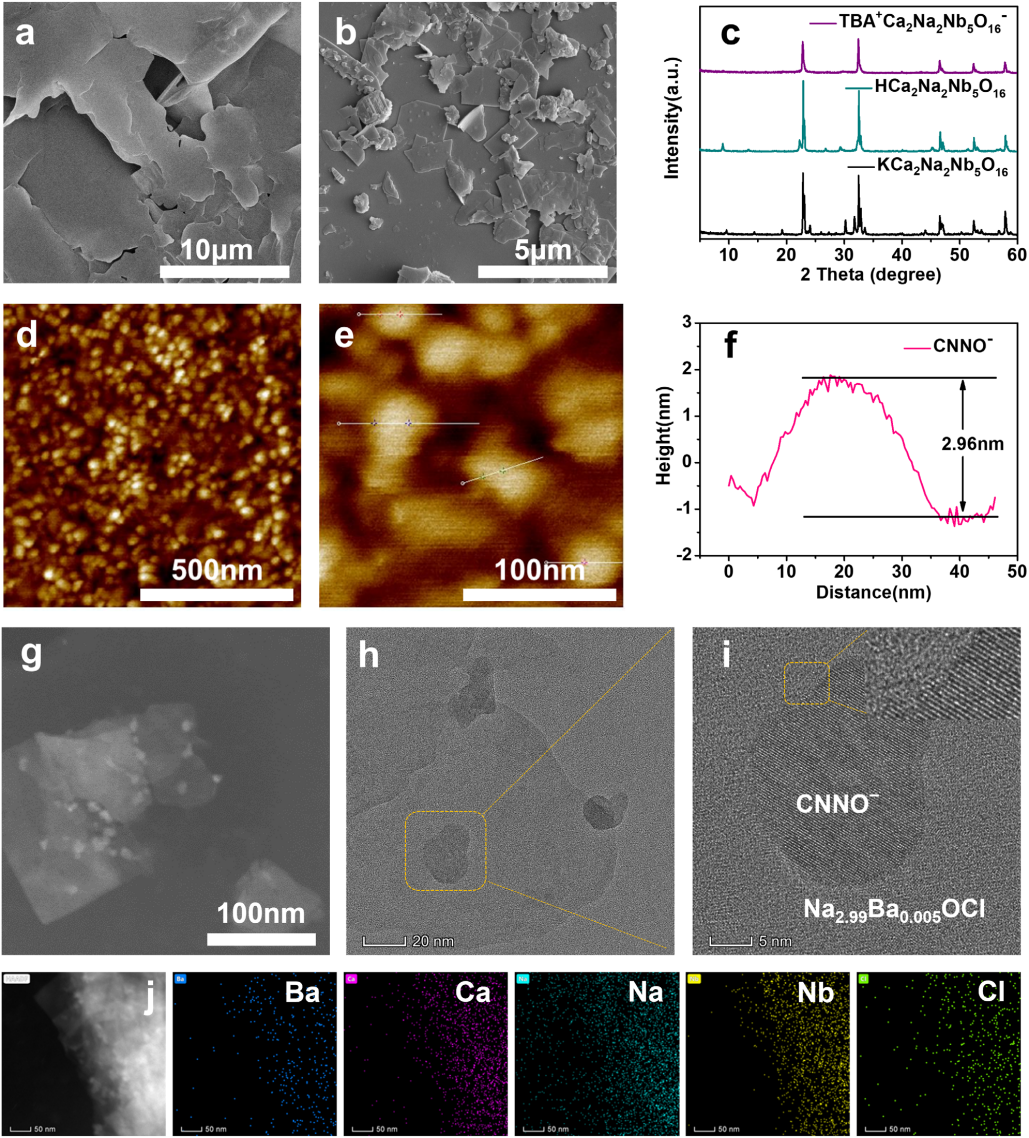
Characterization of Na_2.99_Ba_0.005_OCl/CNNO^‐^ heterostructures. SEM images of a) Na_2.99_Ba_0.005_OCl and b) CNNO^‐^. c) XRD spectra of KCa_2_Na_2_Nb_5_O_16_, HCa_2_Na_2_Nb_5_O_16_, and CNNO^‐^. d,e) AFM images of CNNO^‐^ and f) its corresponding height profile. g,h) TEM images of Na_2.99_Ba_0.005_OCl/CNNO^‐^. i) HRTEM analysis of Na_2.99_Ba_0.005_OCl/CNNO^‐^ nanosheets. j) Corresponding elemental mapping images of Ba, Ca, Na, Nb, and Cl elements.

Scanning electron microscopy (SEM) was used to examine the morphologies of Na_2.99_Ba_0.005_OCl and the CNNO^−^ nanosheets. As shown in **Figure** **2**a, Na_2.99_Ba_0.005_OCl has a 2D lamellar structure. The KCa_2_Na_2_Nb_5_O_16_ before exfoliation displayed a layered structure with stacked nanosheets together (Figure S3, Supporting Information) whereas the exfoliated CNNO^−^ had a dispersed nanosheet structure (Figure 2b). The X‐ray diffraction (XRD) patterns of the starting materials, KCa_2_Nb_3_O_10_ (JCPDS No. 35–1294) and NaNbO_3_ (JCPDS No. 33–1270), presented in Figure S4 (Supporting Information), indicate that the starting materials were formed in a pure phase. Compared with KCa_2_Na_2_Nb_5_O_16_, the XRD pattern of CNNO^−^ after exfoliation revealed the disappearance of the (002), (004), and (005) crystal planes, with diminished peak intensities in the other planes, indicating a loss of 3D dimensionality (Figure 2c). The CNNO^−^ nanosheet thickness was measured using atomic force microscopy (AFM), as shown in Figure 2d,e. The nanosheets predominantly exhibited a lateral size of roughly tens of nanometers and an average thickness of ≈2.96 nm (Figure 2f). This finding is consistent with those of previous studies on other perovskite‐type nanosheets.^[^ ^40^, ^42^ ^]^ TEM images in Figure 2g–i illustrate the microstructure of Na_2.99_Ba_0.005_OCl/CNNO^−^ nanosheet heterostructure. The CNNO^−^ nanosheets adhered closely to the surface of the Na_2.99_Ba_0.005_OCl nanosheets, forming a distinct, layered heterostructure. In the HRTEM images in Figure 2i, two obvious crystal structures were observed: the amorphous structure of Na_2.99_Ba_0.005_OCl and the prominent (110) plane of CNNO^−^ with a lattice spacing of ≈0.28 nm. Moreover, the elemental mapping shown in Figure 2j confirms the presence and consistent distribution of Ba, Ca, Na, Nb, and Cl within the composite material. Unlike crystalline solids, glass comprises atoms and molecules that are not fixed in a rigid lattice and significantly enhances ion mobility by disrupting the ordered structure in crystals through amorphization.^[^ ^25^, ^43^ ^]^ The coupling of glassy ionic conductors and ferroelectric materials fosters the generation of an abundance of mobile Na^+^, thereby enabling efficient ion transport.

**Figure 3:**
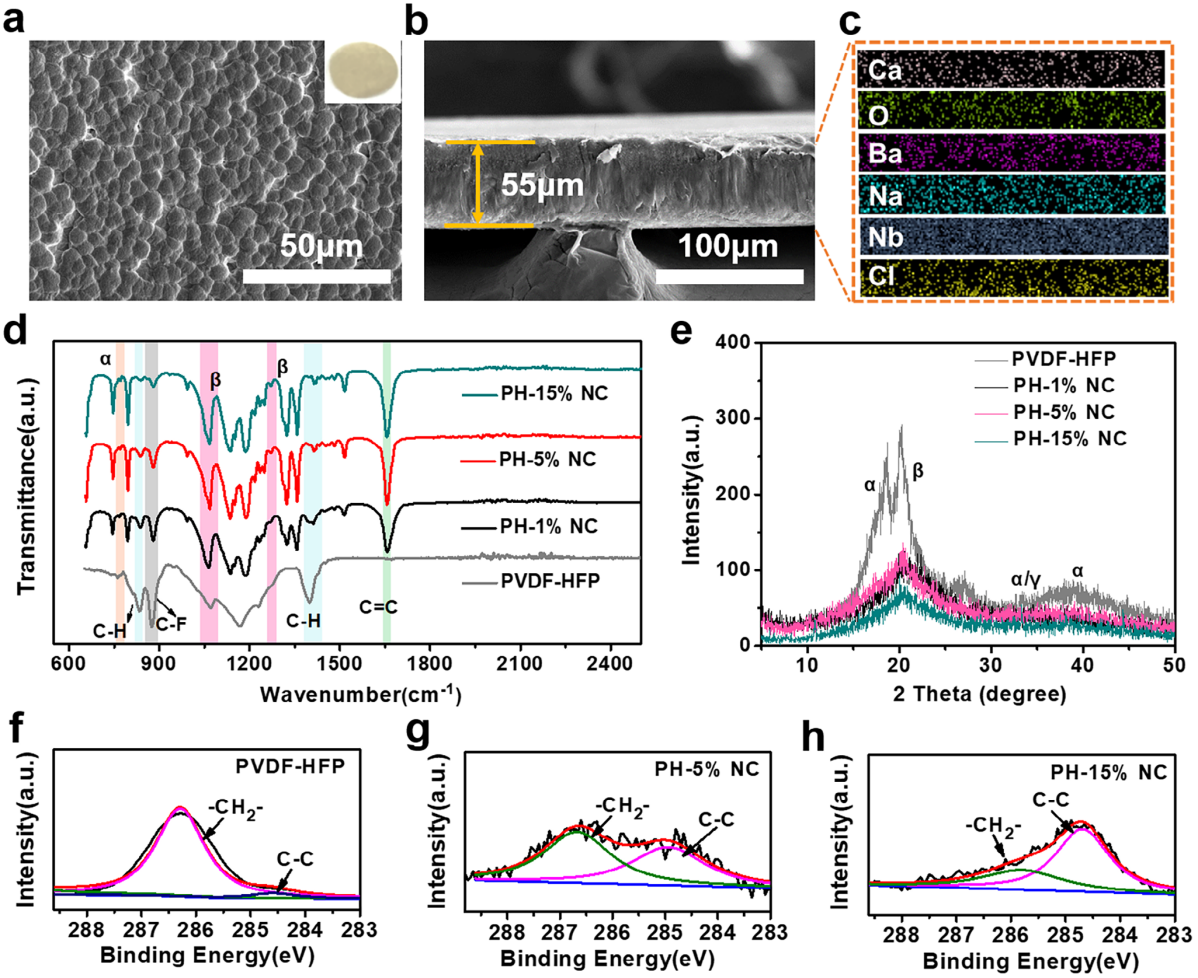
Morphological and structural characterization of metaferroelectrolytes. a) Top view SEM image of PH‐5% NC electrolyte. b) Cross‐sectional SEM image of PH‐5% NC electrolyte, and c) Corresponding energy dispersive spectroscopy (EDS) mappings. d) FTIR spectra and e) XRD spectra of PH‐x% NC and PVDF‐HFP. f–h) XPS spectra of C1s.

Solid metaferroelectrolytes of PH‐x% NC (x = 1, 5, 10, and 15) were obtained by introducing Na_2.99_Ba_0.005_OCl/CNNO^−^ into PVDF‐HFP matrices with different mass contents. Consequently, a comprehensive investigation of the intrinsic properties of PH‐x% NC was conducted to boost the PVDF‐based electrolyte performances through rational optimization of the Na_2.99_Ba_0.005_OCl/CNNO^−^ content. From the PH‐5% NC surface SEM images and elemental mapping, the Na_2.99_Ba_0.005_OCl/CNNO^−^ nanosheets are uniformly distributed in the PVDF‐HFP matrix, exhibiting a relatively even, uniform surface (**Figure** **3**a; Figure S5 Supporting Information). From the cross‐sectional SEM image, the PH‐5% NC electrolyte thickness appeared to be ≈55 µm, and the corresponding element scanning images confirmed that the ion transport channel construction in the normal direction within the electrolyte membrane enhanced ion migration between the anode and cathode (Figure 3b,c). The dehydrofluorination and cross‐linking mechanisms are further discussed in light of the Fourier transform infrared (FT‐IR) spectra shown in Figure 3d. The absorption bands at 840 and 1 402 cm^−1^ can be assigned to the –CH_2_ group whereas the peak at 876 cm^−1^ corresponds to the –CF_2_ group.^[^ ^44^ ^]^ The PH‐x% NC (x = 1, 5, or 15) electrolytes exhibited new stretching vibration peaks at 1 657.2 cm^−1^ associated with the C = C bond. With increased Na_2.99_Ba_0.005_OCl/CNNO^−^ content, the –CH_2_ and –CF_2_ peak intensities decreased, indicating that hydrogen and fluorine atoms detached under attack by the alkaline group Na_2.99_Ba_0.005_OCl. Conversely, the C = C absorption peak gradually increased, possibly related to the crystallinity decrease and new C = C bond formation. The characteristic vibrational peaks at 1 068,1 275, and 763 cm^−1^ were ascribed to the polar β and α phases. Further Na_2.99_Ba_0.005_OCl/CNNO^−^ addition increased the intensity of the ferroelectric β phase; moreover, the α phase slowly diminished. This result demonstrated the Na_2.99_Ba_0.005_OCl‐induced phase transformation of the PVDF‐HFP matrices from the α to β phases. XRD analysis verified this phase transition, as shown in Figure 3e. The 18.5 and 20.5° peaks corresponded to the (020) plane of the α and (110) plane of the β phase, respectively.^[^ ^45^, ^46^ ^]^ As can be seen, the α phase intensity was reduced by introducing Na_2.99_Ba_0.005_OCl/CNNO^−^, which was beneficial for forming the polar β phase. The β phase was highly polar, so the strong interactions between the β phase and Na^+^ could induce uniform redistribution of the sodium ion flux. Combined with the XPS pattern in Figure 3f–h, where a distinct carbon chain peak emerged at 284.5 eV, these results conclusively demonstrate the formation of a cross‐linked network within the PVDF‐based solid electrolytes.^[^ ^47^ ^]^ Excessive Na_2.99_Ba_0.005_OCl/CNNO^−^ leads to a higher degree of polymer carbonization.

**Figure 4:**
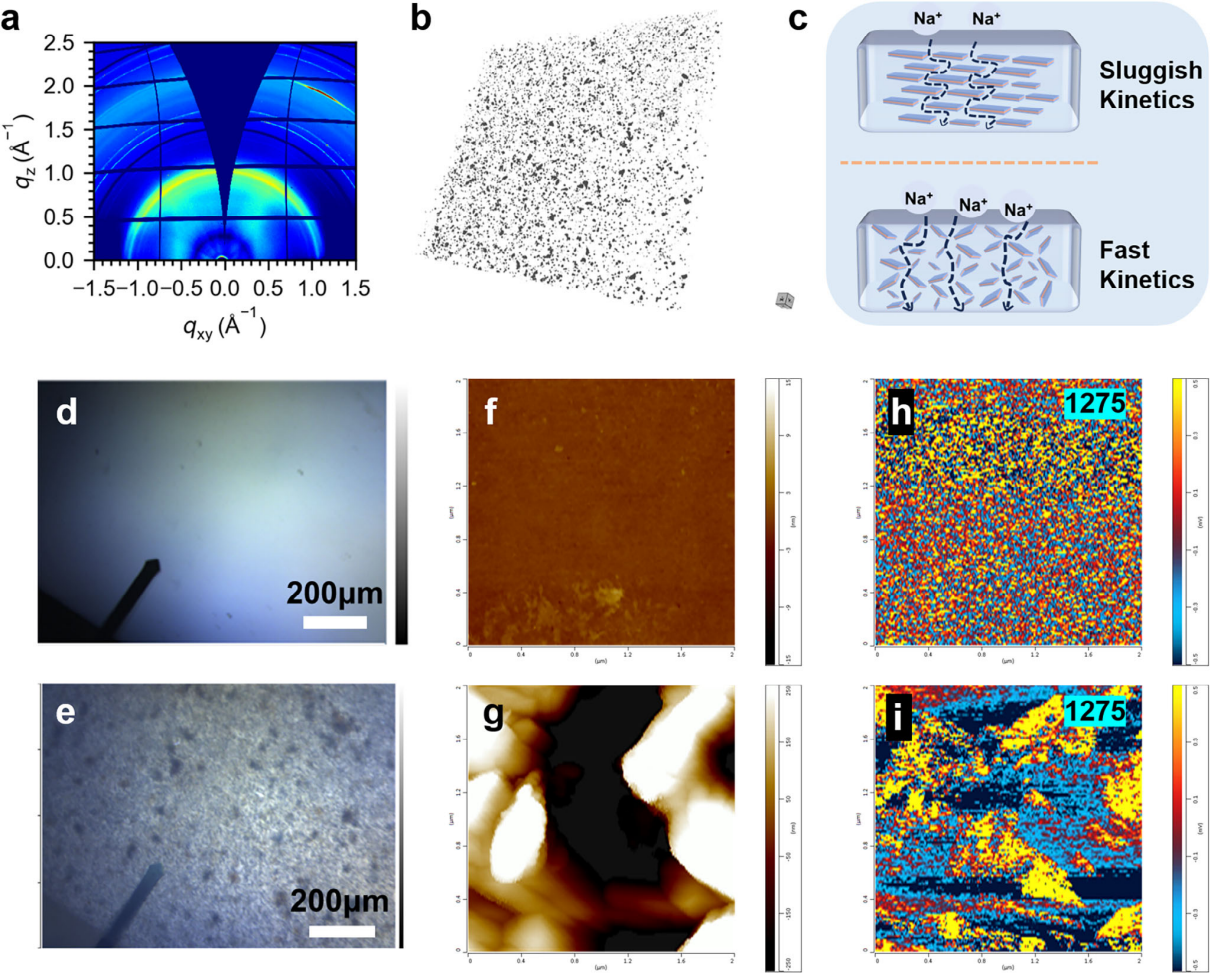
The organization of nanosheets within the metaferroelectrolyte membrane and the phase transitions of PVDF‐HFP. a) Typical 2D‐GIWAXS image and b) 3D reconstruction collected with XCT of PH‐5% NC. c) Comparison of sodium ion transfer of internally ordered electrolytes and proposed cross‐linked electrolytes. Optical images of d) PVDF‐HFP and e) PH‐5% NC. AFM images of f) PVDF‐HFP and g) PH‐5% NC (2 µm × 2 µm). AFM‐IR chemical mapping at 1 275 cm^−1^ of h) PVDF‐HFP and i) PH‐5% NC (2 µm × 2 µm).

To explore the 2D/2D nanosheet orientation distribution in polymeric substrates, we conducted grazing‐incidence wide‐angle X‐ray scattering (2D‐GIWAXS), as illustrated in **Figure** **4**a. The PH‐5% NC membrane did not reflect strongly along the q_z_ direction and presented rings with uniform intensities. This indicates that the nanosheet distribution within the polymer exhibited a nonspecific arrangement orientation and that the arrangement direction had a certain degree of randomness.^[^ ^48^, ^49^ ^]^ Similar to the disordered arrangement of powdered nanosheets (Figure S6, Supporting Information), the polymer molecular chains did not change the original nanosheet orientations. This agrees with the X‐ray computed tomography (XCT) images shown in Figure 4b. The 3D construction of the PH‐5% NC membrane was an insightful cue for the nanosheet distribution information, in line with the GIWAXS measurements. The XCT images also reveal the cross‐sectional and surface views of PH‐5% NC in Figure S7b,c (Supporting Information), accentuating their internal uniformity, similar to a dilute or trace nanocomposite electrolyte with a quasi‐homogeneous in situ solidification. In comparison, the PH‐15% NC exhibited stratification (Figure S7d–f, Supporting Information), where excessive cross‐linking during the polymer film formation process caused the nanosheets to aggregate unidirectionally, resulting in discontinuous ion transport channels that slowed down the ion conduction rate. In contrast to conventional solid electrolytes featuring ordered arrangements, the PH‐5% NC electrolyte prepared through partial cross‐linking offers numerous 2D transport pathways for sodium ions, fostering faster sodium ion transfer kinetics, along with the heterostructure interface ferroelectric field induced ionic transport (Figure 4c). AFM‐IR was employed to analyze the morphology and directly map the polar conformational distribution in Figure 4d–i. Integrating AFM with wavelength‐tunable lasers provides nanoscale infrared chemical maps for detecting microstructures.^[^ ^50^ ^]^ Figure 4d,e presents the PVDF‐HFP and PH‐5% NC membrane surfaces under optical microscopy. Notably, compared to the PVDF‐HFP membrane, the PH‐5% NC membrane surface was covered with uniformly distributed 2D/2D nanosheets. Thus, there was a significant difference in surface heights (Figure 4f,g). Meanwhile, IR chemical mapping of the polar β phase was tested at 1 275 cm^−1^, as shown in Figure 4h,i. The chemical patterns of PVDF‐HFP and PH‐5% NC were substantially different. Chemical cross‐linking induced extensive aggregation of the polar β phase in PH‐5% NC, indicating higher electroactivity. By contrast, the α phase absorbance at 976 cm^−1^ lessened, as depicted in Figure S8 (Supporting Information). This result is consistent with that shown in Figure 3.

### 2.3 Influence of Ferroelectric Field on Sodium Ion Transport

**Figure 5:**
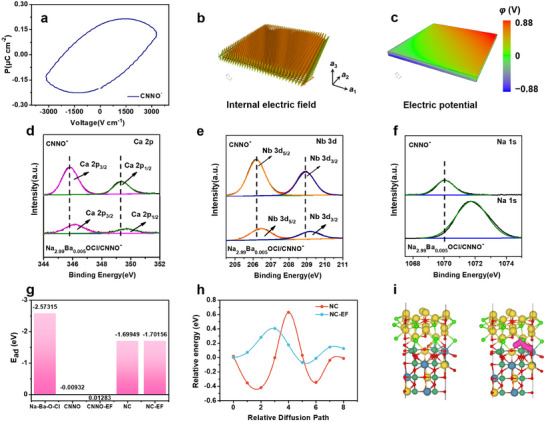
Ferroelectric potential/EF distribution simulation and ionic coupling DFT calculation. a) Ferroelectric responses (P‐E curve) of CNNO^‐^. b) Simulation of internal EF and c) Corresponding intrinsic ferroelectric potential generated by polarization. XPS spectra of d) Ca 2p, e) Nb 3d, and f) Na 1s of CNNO^‐^ and Na_2.99_Ba_0.005_OCl/CNNO^‐^, respectively. g) Sodium ion adsorption energies on different materials. h) Calculated energy barrier of sodium ion with/without ferroelectric field. i) Schematic diagram of ion migration for Na_2.99_Ba_0.005_OCl/CNNO^‐^ heterostructure.

A P‐E loop and software simulations were performed to estimate the positive effect of the intrinsic CNNO^−^ ferroelectricity on the sodium ion kinetics. **Figure** **5**a shows an exquisite hysteresis response, crucial for ferroelectricity manifestation from the P‐E loop.^[^ ^40^, ^41^ ^]^ Figure 5b illustrates the internal EF due to spontaneous polarization and the corresponding potential generated (Figure 5c). The simulations provide evidence that spontaneous polarization causes the ferroelectric domains to reorient, yielding a tilted potential gradient. Using these parameters, the calculated ferroelectric potential intensity was ≈12.44 mV nm^−1^, which acted as an additional accelerator to improve the sodium ion kinetics. X‐ray photoelectron spectroscopy (XPS) was used to determine the elemental compositions and chemical statuses of Na_2.99_Ba_0.005_OCl/CNNO^−^ and CNNO^−^. Cl, Ca, Na, Nb, and O were confirmed from the survey spectra shown in Figure S9 (Supporting Information). The Ca 2p spectrum in Figure 5d, located at ≈345.9 and 349.5 eV, could be separately attributed to Ca 2p_3/2_ and Ca 2p_1/2_, respectively.^[^ ^51^ ^]^ The high‐resolution XPS spectrum of Nb 3d, as shown in Figure 5e, was divided into two peaks: Nb 3d_5/2_ at 206.6 eV and Nb 3d_3/2_ at 209.3 eV.^[^ ^52^ ^]^ The main peak at 1 070.3 eV corresponded to the Na 1s binding energy (Figure 5f). Notably, the Ca 2p, Nb 3d, and Na 1s peaks of Na_2.99_Ba_0.005_OCl/CNNO^−^ were shifted to higher binding energies than those of CNNO^−^, suggesting the presence of strong interfacial interactions and charge transfer between Na_2.99_Ba_0.005_OCl and CNNO^−^. To understand the positive effect of the self‐built‐in EF more quantitatively, we conducted a comprehensive comparative analysis of the sodium ion adsorption and diffusion behavior using density functional theory (DFT) calculations. Figure 5g displays the adsorption energies of Na atoms in the surface layer of Na_2.99_Ba_0.005_OCl, CNNO^−^, and the interface layer of Na_2.99_Ba_0.005_OCl/CNNO^−^. The corresponding adsorption energies on CNNO^−^ and Na_2.99_Ba_0.005_OCl/CNNO^−^ are also listed with an external EF for comparison. Consequently, Na_2.99_Ba_0.005_OCl presented the strongest adsorption energy (2.57 eV) toward the Na atom. In contrast, CNNO^−^ had little adsorption capacity toward Na atoms, and the result was not significantly different from the EF influence. Specifically, the heterostructure construction substantially decreased the adsorption energies by 1.69 eV, and the EF had a similarly limited impact. This result suggests that the coupled Na_2.99_Ba_0.005_OCl can spontaneously adsorb nearly free sodium ions at the interface and accelerate ion migration through the intrinsic ferroelectric field of CNNO^−^. Furthermore, Figure 5h shows the influence of the inherent ferroelectric field on ion migration by analyzing the diffusion barriers. Notably, the sodium ion diffusion barriers in Na_2.99_Ba_0.005_OCl/CNNO^−^ were significantly reduced under the same diffusion path when EF was considered, revealing the greatly enhanced diffusion kinetics of sodium ions in the ferroelectric effect. Hence, based on DFT calculations, we explored the mechanism of action of Na_2.99_Ba_0.005_OCl/CNNO^−^ nanosheets in polymer matrices, including creating inherent ferroelectric fields as “acceleration zones” for highly efficient ion transport. The front view of ion migration in the heterostructures (Figure 5i) and the side view of the structures of Na atoms adsorbed on different materials are displayed in sequence (Figure S10a–e, Supporting Information).

### 2.4 Electrochemical and Mechanical Properties of the PH‐5% NC Metaferroelectrolyte

**Figure 6:**
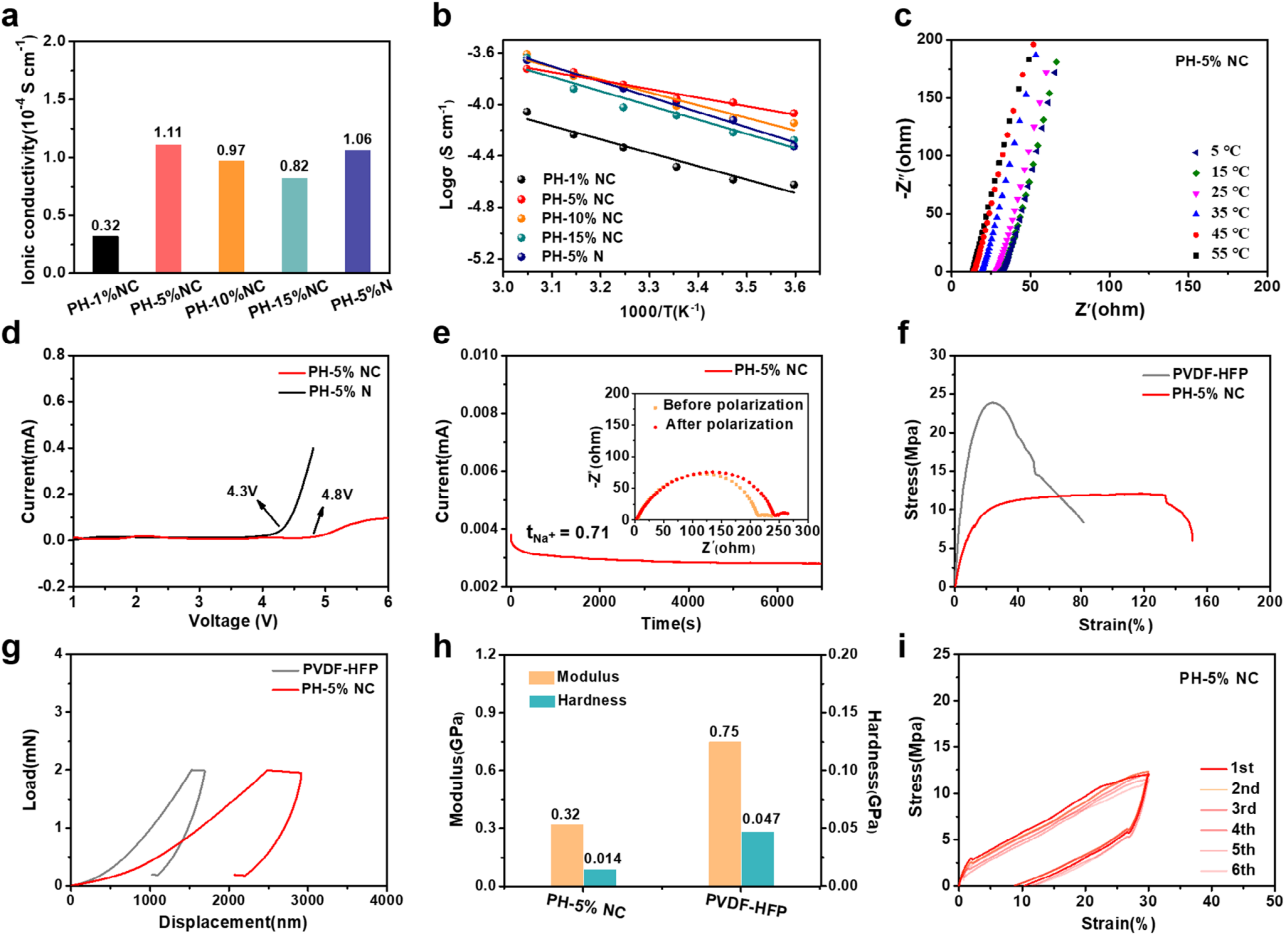
The electrochemical and mechanical properties of the PVDF‐based metaferroelectrolytes. a) Ionic conductivities of PVDF‐based electrolytes at 25 °C. b) Arrhenius plots of PVDF‐based electrolytes. c) EIS spectra of SS/PH‐5% NC/SS cell from 5 to 55°C. d) LSV curves of PH‐5% NC and PH‐5% N electrolyte. e) Current‐time curve of PH‐5% NC electrolyte. f, g) Stress–strain curves and load‐displacement curves of PVDF‐HFP and PH‐5% NC electrolyte. h) Its corresponding elastic modulus and hardness. i) Cyclic stress‐strain curves with the elongation of 30% for PH‐5% NC electrolyte.

The electrochemical properties of the PVDF‐based metaferroelectrolytes served as selection criteria for solid‐state electrolyte applications. Compared with the PH‐1% NC electrolyte, the ionic conductivities of the PH‐x% NC electrolytes (5, 10, and 15 wt.%) and the PH‐5% N electrolyte increased, as shown in **Figure** **6**a. The ionic conductivity of PH‐5% NC metaferro‐ electrolyte was optimal (1.11 × 10^−4^ S cm^−1^ at 25 °C). The non‐monotonic behavior observed in the ionic conductivity can be attributed to the excessive nanosheet filling in the PVDF‐HFP matrix, leading to the agglomeration and layering of the nanosheets, thereby decreasing the ion conductivity. In addition, the ionic conductivity of the PH‐5% NC electrolyte was higher than that of the PH‐5% N electrolyte. Temperature‐dependent ion conductivity can be improved by increasing temperature (from 5 to 55 °C) and following the Arrhenius equation (Figure 6b).^[^ ^53^ ^]^ The activation energy required for ion transport in the PH‐5% NC electrolyte was the lowest (0.13 eV), and ion conduction was the fastest among the composite electrolytes, as displayed in Figure S11 (Supporting Information). Figure 6c and Figure S12 (Supporting Information) show the impedance spectra of the composite electrolytes. The PH‐5% NC electrolyte exhibited an oxidation stability exceeding 4.8V, much higher than that of the PH‐5% N electrolyte (≈4.3 V), as illustrated in Figure 6d. The high stability of the electrolyte membrane makes it a promising candidate for a high‐voltage cathode material. Benefiting from the 2D heterostructures and ferroelectric effect of CNNO^−^, the improved transference number (t _Na+_) of PH‐5% NC was evaluated as 0.71 (Figure 6e), as compared to that of PH‐5% N (0.56; Figure S13, Supporting Information). This phenomenon can be explained by the 2D heterostructures disrupting the orderly arrangement of the polymer matrix and forming an interconnected ion‐conductive network, which significantly enhanced the swift‐hopping transport of ions along the cross‐linked molecular chains and 2D nanosheets. In addition, the presence of a built‐in EF promotes the movement of free Na^+^. The differential scanning calorimetry (DSC) curves in Figure S14 (Supporting Information) indicated that the glass transition temperature (T_g_) of PH‐5% NC electrolyte has shifted to lower temperatures (62.8 °C) compared to pure PVDF‐HFP (91.3 °C).^[^ ^54^ ^]^ This result proves that introducing Na_2.99_Ba_0.005_OCl/CNNO^−^ nanosheets effectively reduced the crystallinity of PVDF‐HFP. As observed in the stress‐strain curves (Figure 6f), strong cross‐linking interactions enhanced the flexibility of the PH‐5% NC. Its mechanical strain, 133.2%, was much higher than that of pure PVDF‐HFP (23.4%), which was conducive to forming better interfacial contact.^[^ ^55^ ^]^ The tensile strength of the PH‐5% NC electrolyte reached 12.1 MPa, which is beneficial for suppressing sodium dendrite penetration. As the Na_2.99_Ba_0.005_OCl/CNNO^−^ nanosheets increased, the tensile strength decreased, and the stretchability improved, as shown in Figure S15 (Supporting Information). Thus, the PH‐5% NC electrolyte exhibited excellent comprehensive mechanical performance.

Meanwhile, according to the depth difference between the lifting curve and the loading curve under the same loading force (Figure 6g), it can be found that the cross‐linking PH‐5% NC exhibits better elasticity than PVDF‐HFP. Specifically, as depicted in Figure 6h, the Young's modulus of PH‐5% NC electrolyte was 0.32 GPa, lower than that of PVDF‐HFP (0.75 GPa). Furthermore, the hardness of PH‐5% NC electrolyte, which was 0.014 GPa, was lower than that of PVDF‐HFP (0.047 GPa). In addition, the cyclic stress‐strain tests were performed to assess electrolyte membrane flexibility in Figure 6i. The PH‐5% NC electrolyte showed remarkable elasticity, as evidenced by the overlapping stress‐strain curves during six consecutive cycles with an elongation of 30%. The improved intrinsic elasticity observed in PH‐5% NC electrolyte was achieved through precise cross‐linking of PVDF‐HFP chains. Compared to poor interface contact and increased interfacial resistance between electrolyte and electrodes for traditional solid‐state electrolyte battery, the modulus reduction of the ferroelectric PVDF‐HFP network increases the electrolyte elasticity and promotes the rough and resilient conformal interfacial contact recovery between the metaferroelectrolytes and electrodes during charge‐discharge cycling.

### 2.5 Dendrite Suppression Capability and Flame Retardancy Testing

**Figure 7:**
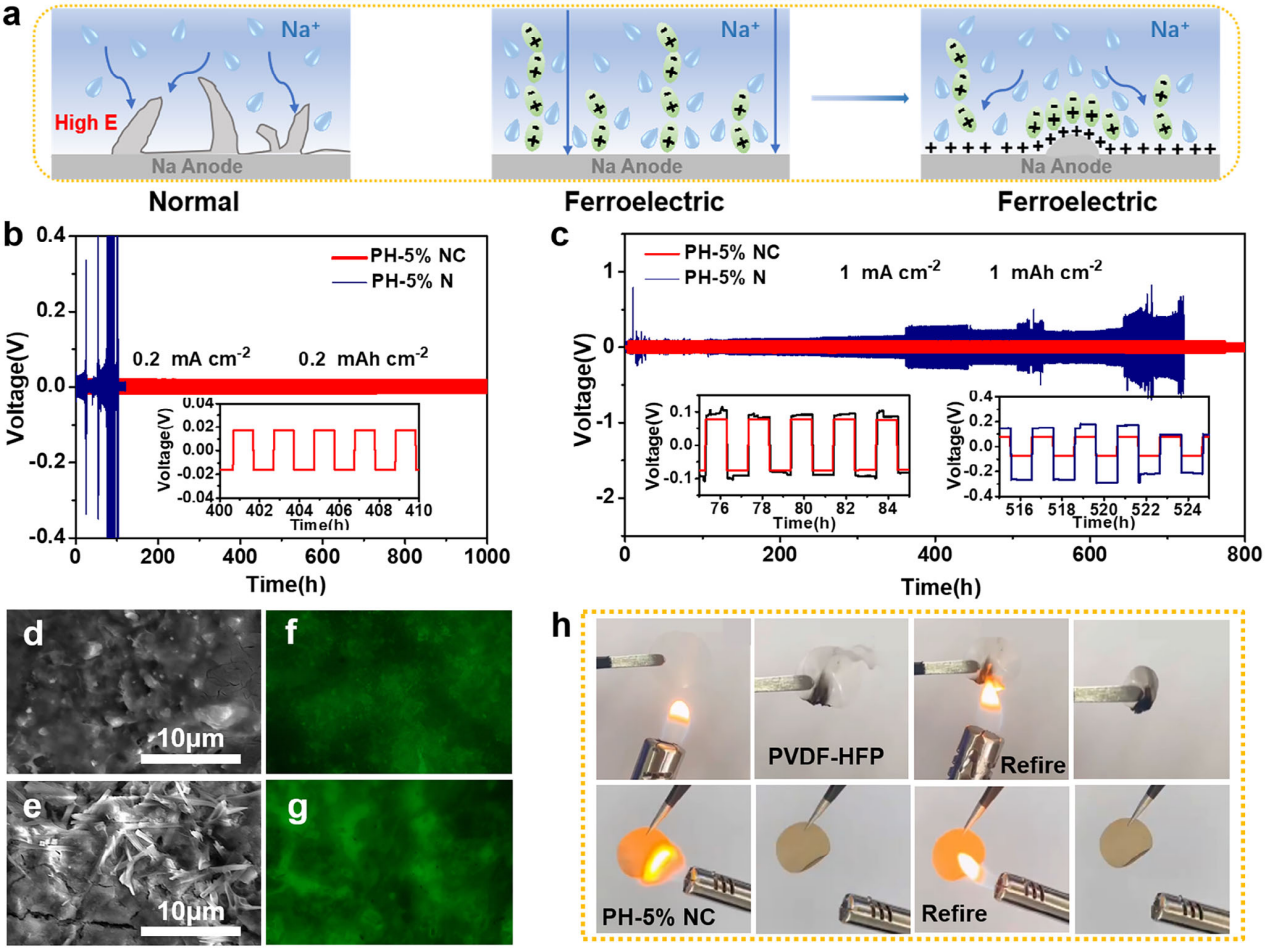
Dendrite suppression capability and flame retardancy testing. a) Mechanism schematic of sodium ion transport and deposition regulation. Voltage profiles of continued sodium plating/stripping cycling of PH‐5% NC and PH‐5% N electrolytes at a current density of b) 0.2 mA cm^−2^ and c) 1 mA cm^−2^ at 25 °C. SEM images of cycled Na anode surfaces using d) PH‐5% NC and e) PH‐5% N electrolyte. Fluorescence images of Na surfaces after cycling with f) PH‐5% NC and g) PH‐5% N electrolyte. h) Combustion tests of PVDF‐HFP and PH‐5% NC electrolyte.

The mechanism by which PH‐5% NC metaferroelectrolyte actively suppressed dendrite formation is proposed in **Figure** **7**a.^[^ ^38^, ^56^ ^]^ First, with the assistance of the electrostatic field inside the battery, the ferroelectric layered Na_2.99_Ba_0.005_OCl/CNNO^−^ exhibits a large spontaneous polarization, which effectively promotes rapid sodium ion transport at the interface, thereby reducing the sodium ion concentration gradient near the sodium anode. During Na deposition, the local potential near the protrusions increases because of the microscopic roughness of the anode surface. Under the action of a strong potential, sodium ions are preferentially deposited in the protuberant tip area, producing sharp sodium dendrites. Finally, dendrite pressure on the ferroelectric electrolyte causes the CNNO^−^ domain dipole‐induced residual polarization to produce a voltage around the dendrite tips in the direction opposite from the potential, driving the ions to redistribute to the flat region and form a flat sodium metal surface. To emphasize the significant role of ferroelectric heterostructure nanosheets in the compatibility and stability of sodium metal / electrolyte interfaces, Na|PH‐5% NC|Na symmetric cells were tested at 25 °C. As shown in Figure 7b, the long‐term cycling performance of the Na|PH‐5% NC|Na symmetric battery was measured at 0.2 mA cm^−2^. The Na|PH‐5% NC|Na symmetric battery maintained a stable plating/stripping plateau after 1 000 h. For comparison, the Na|PH‐5%N|Na symmetric battery exhibited substantial voltage fluctuation, causing a short‐circuit phenomenon after 100 h owing to dendrite puncture. In particular, when the current was set to1 mA cm^−2^ in each cycle (Figure 7c), the voltage of the Na|PH‐5% NC|Na symmetric battery remained stable, with a voltage polarization of ≈75 mV, compared to the fluctuating polarization of the Na|PH‐5% N|Na symmetric battery. Voltage polarization may be ascribed to irreparable damage to the sodium metal/electrolyte interfaces during the sodium plating/stripping process. Given the above results, the intrinsic elasticity of the PH‐5% NC electrolyte can adapt to the volume variations of sodium in electroplating/stripping process, meanwhile the Na_2.99_Ba_0.005_OCl/CNNO^−^ nanosheets with ferroelectric properties possess the ability to regulate sodium deposition and maintain the stability of the sodium/electrolyte interface. The morphologies of the sodium dendrites on the cycled sodium surfaces were observed using SEM. As illustrated in Figure 7d,e, the cycled sodium surfaces of the Na|PH‐5% NC|Na battery displayed a flat, dense morphology. In contrast, the cycled sodium surfaces of the Na|PH‐5% N|Na battery exhibited obvious sodium dendrites, confirming the crucial role of PH‐5% NC in inhibiting dendrite growth.^[^ ^57^ ^]^ Fluorescent probes represent a new method for studying sodium plating.^[^ ^58^ ^]^ The phenolic sites of the 4‐(1,2,2‐triphenylvinyl) phenol (TPEOH) probe can rapidly react with active sodium to induce a fluorescence switch that does not change the original structure of the sodium anode surface. Therefore, the fluorescence images detected by TPEOH accurately show the condition of the sodium anode, as shown in Figure 7f,g. After spraying an anhydrous ether solution of TPEOH on the sodium surface after cycling, the cycled sodium metal with PH‐5% NC showed uniformity in the fluorescence images. In contrast, the fluorescence concentration and intensity of the cycled sodium metal with PH‐5% N increased, suggesting the formation of many sodium dendrites. The fluorescence images are consistent with the SEM images. The flame retardancy of the PH‐5% NC electrolyte was evaluated using a combustion test. As illustrated in Figure 7h, the intrinsic superior thermal resistance of PVDF‐HFP endowed both PVDF‐HFP and the PH‐5% NC with repeatable self‐extinguishing behavior. In contrast, the PVDF‐HFP suffered from significant melt deformation whereas the introducing of strongly ferroelectric‐coupled Na_2.99_Ba_0.005_OCl/CNNO^−^ heterostructures significantly enhanced the thermal stability of the polymer matrix. This structure enables PH‐5% NC metaferroelectrolyte to simultaneously achieve exceptional flame retardancy and shape retention, thereby improving solid‐state sodium metal batteries safety.

### 2.6 Electrochemical Properties of Na_3_V_2_(PO_4_)_3_//Na Solid‐State Metaferroelectrolyte Batteries

**Figure 8:**
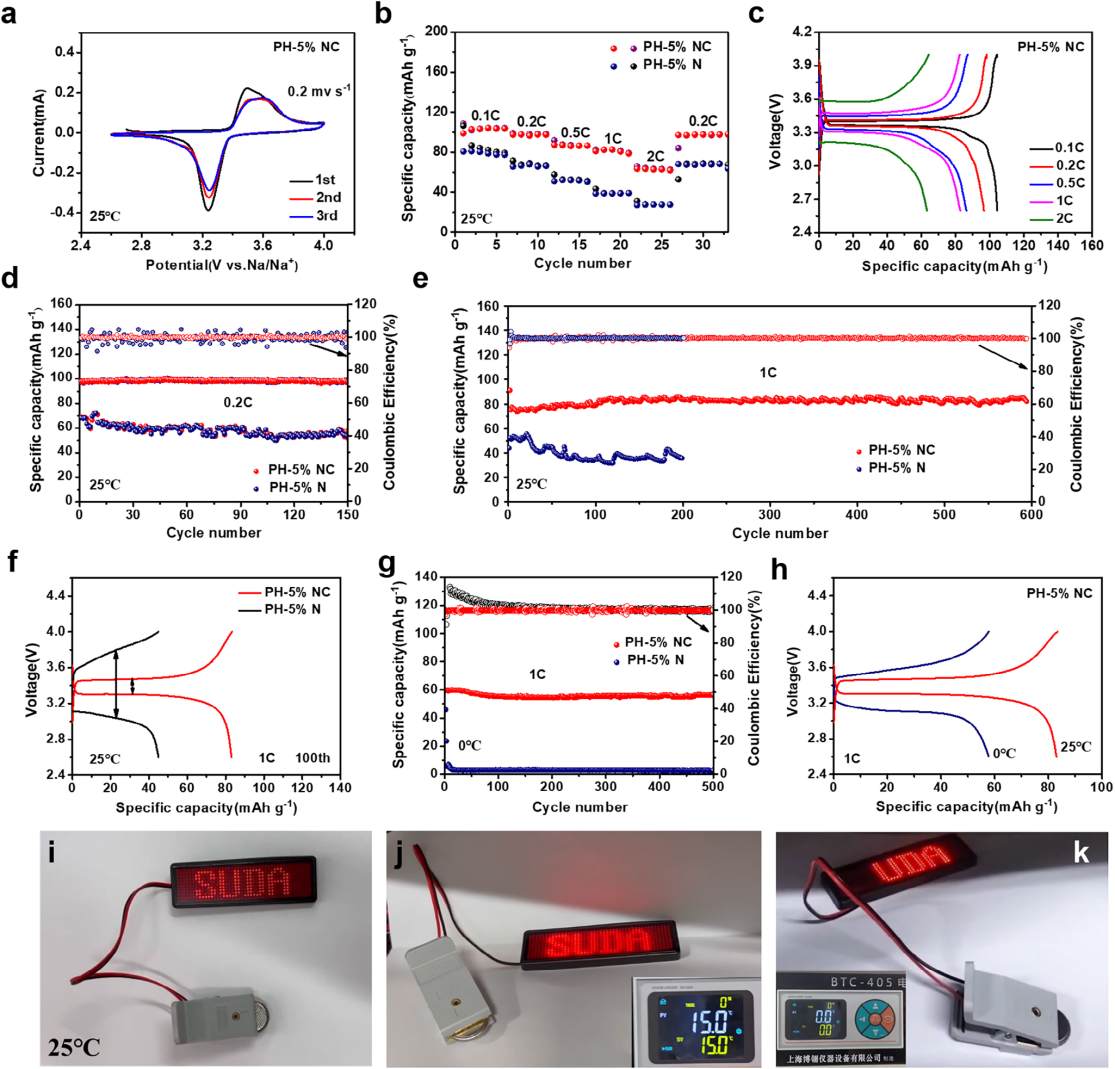
Electrochemical properties of Na_3_V_2_(PO_4_)_3_//Na solid‐state metaferroelectrolyte batteries. a) Cyclic voltammogram curves of Na_3_V_2_(PO_4_)_3_/PH‐5%NC/Na solid‐state battery at 25 °C. b) Rate performances of Na_3_V_2_(PO_4_)_3_//Na cells at 25 °C. c) Charge‐discharge curves of Na_3_V_2_(PO_4_)_3_/PH‐5%NC/Na cell at various rates at 25 °C. d) Cycle performances of Na_3_V_2_(PO_4_)_3_//Na cells at 0.2 C at 25 °C. e) Long‐term cycling stability of Na_3_V_2_(PO_4_)_3_//Na cells at 1 C and 25 °C. f) Charge‐discharge curves of Na_3_V_2_(PO_4_)_3_//Na cells after 100 cycles at 1 C and 25 °C. g) Long‐term cycling stability of Na_3_V_2_(PO_4_)_3_//Na cells at 1 C and 0 °C. h) Charge‐discharge profiles of Na_3_V_2_(PO_4_)_3_/PH‐5%NC/Na cell at 1 C and 25 and 0 °C. Digital photos of LED illuminated by Na_3_V_2_(PO_4_)_3_/PH‐5%NC/Na coin cell at i) 25, (j) 15, and (k) 0 °C.

To evaluate the stability and electrochemical properties of the PH‐5% NC metaferroelectrolyte in solid‐state sodium metal batteries at room temperature, Na_3_V_2_(PO_4_)_3_//Na batteries were assembled. Cyclic voltammetry (CV) curves showed that the Na_3_V_2_(PO_4_)_3_/PH‐5% NC/Na battery possessed superior redox reaction reversibility in **Figure** **8**a, suggesting that PH‐5% NC had remarkable interfacial stability with both the Na_3_V_2_(PO_4_)_3_ cathode and Na metal anode. Conversely, the CV curves of the PH‐5% N exhibited significantly enhanced polarization in Figure S16 (Supporting Information). Furthermore, distinct oxidative instability was observed during the third charging cycle, further illustrating the superior electrode‐electrolyte interfacial stability of the PH‐5% NC. As shown in Figure 8b, the Na_3_V_2_(PO_4_)_3_/PH‐5% NC/Na cell exhibited outstanding rate performance at various rates. The specific capacities of the Na_3_V_2_(PO_4_)_3_/PH‐5% NC/Na cell were 103.4, 97.9, 87.1, 82.7, and 62.8 mAh g^−1^ at 0.1, 0.2, 0.5, 1, and 2 C, respectively, and when returning to 0.2 C, the capacity recovered. This was significantly higher than those of the Na_3_V_2_(PO_4_)_3_/PH‐x% NC/Na (x = 1,10,15) cell in Figure S17a (Supporting Information). Figure 8c shows the corresponding galvanostatic charge‐discharge (GCD) profiles of the Na_3_V_2_(PO_4_)_3_/PH‐5% NC/Na cell, illustrating the excellent interfacial stability and reversibility of the electrochemical reaction. The cycling performance of the Na_3_V_2_(PO_4_)_3_/PH‐5% NC/Na cell at 0.2 C is shown in Figure 8d. Surprisingly, even after 150 cycles, the discharge capacity was still as high as 98.7 mAh g^−1^ with 99.8% capacity retention, compared to 78.3% for the Na_3_V_2_(PO_4_)_3_/PH‐5% N/Na cell. Figure 8e displays the long cycling stability of Na_3_V_2_(PO_4_)_3_/PH‐5% NC/Na battery at 1 C. It still possessed an outstanding reversible capacity of 83.7 mAh g^−1^ with 100% Coulombic efficiency after 600 cycles. In contrast, the Na_3_V_2_(PO_4_)_3_/PH‐5% N/Na cell exhibited lower capacity and rapidly decayed after 40 cycles under the same conditions. A comparison of the cycling stability of the other electrolytes in Figure S17b,c (Supporting Information) demonstrates that the PH‐5% NC metaferroelectrolyte was optimal among the composite electrolytes. Figure S18 (Supporting Information) further indicates that the Na_3_V_2_(PO_4_)_3_/PH‐5% NC/Na battery still has a smooth charge‐discharge platform after 500 cycles, revealing the long‐term effectiveness of the interface between the electrolytes and the electrodes. Notably, the Na_3_V_2_(PO_4_)_3_/PH‐5% NC/Na battery demonstrated exceptional high‐rate capability and interfacial stability during fast charge‐discharge processes, maintaining a reversible capacity of 29.6 mAh g^−1^ with 100% Coulombic efficiency over 500 cycles at 5 C in Figure S19 (Supporting Information). To evaluate the practical applicability of flexible PH‐5% NC, we have estimated that solid‐state sodium metal batteries possess a mass energy density of ≈190.6 Wh kg^−1^ and a volumetric energy density of ≈306.9 Wh L^−1^, as detailed in Supporting Information.^[^ ^59^, ^60^, ^61^ ^]^ Notably, by precisely refining the thickness of the flexible electrolyte membrane to just 25 µm, we can elevate the energy density to over 600 Wh L^−1^, vastly surpassing the current commercial sodium‐ion liquid‐electrolyte batteries (365 Wh L^−1^). In addition, the PH‐5% NC membrane density was 1.91 g cm^−3^, which was lower than that of Na_3_OCl electrolytes^[^ ^62^ ^]^ (≈2.2 g cm^−3^) and conventional oxide‐based electrolytes^[^ ^63^ ^]^ (≈5.1 g cm^−3^), that was more conducive to enhancing the specific energy density of the battery system. Moreover, the PH‐5% NC‐based battery exhibited lower polarization than the PH‐5% N‐based battery, as shown in Figure 8f, which is consistent with the electrochemical impedance spectroscopy (EIS) spectra (Figure S20, Supporting Information). The interface impendence of the PH‐5% NC‐based battery was 222.5 Ω, smaller than that of the PH‐5% N‐based battery (324.9 Ω), confirming the positive effect of the ferroelectric heterostructure nanosheets. Although the battery exhibited excellent cycling performance at room temperature, there were still some challenges in its operation at low temperatures. We attempted to conduct long‐term cycling stability tests at 1 C for the PH‐5% NC‐based battery at 0 and 15 °C, as shown in Figure 8g and Figure S21 (Supporting Information). Notably, desirable capacities of 73.1 mAh g^−1^ and 56.4 mAh g^−1^ were retained after 500 cycles at 15 and 0 °C, respectively. Surprisingly, the PH‐5% NC‐based battery exhibited stable charge‐discharge performance even at − 20 °C and 0.1 C (Figure S22, Supporting Information), manifesting its strong potential even under low‐temperature conditions. As shown in Figure 8h, the Na_3_V_2_(PO_4_)_3_/PH‐5% NC/Na cell displayed a higher polarization voltage at 0 °C than at 25 °C due to the slower sodium ion migration and the poor interface compatibility at low temperatures. We assembled a Na_3_V_2_(PO_4_)_3_/PH‐5% NC/Na coin cell to light up an LED panel at 25, 15, and 0 °C. Figure 8i–k shows that an LED panel with a voltage above 3 V could be powered even at 0 °C, demonstrating the cell's practicability in low‐temperature electronic devices.

To assess the application potential of the PH‐5% NC metaferroelectrolyte in solid‐state batteries at room temperature, Na_3_V_2_(PO_4_)_3_/PH‐5% NC/HC full cells were assembled. Figure S23 (Supporting Information) presented the cycling performance and corresponding GCD profiles of the Na_3_V_2_(PO_4_)_3_/PH‐5% NC/HC full cell at 1C. The full cell maintained an impressive reversible capacity of 47.3 mAh g^−1^ with 99.6% Coulombic efficiency over 120 cycles, while the GCD profiles further confirmed the exceptional interfacial stability and electrochemical reversibility of the PH‐5% NC metaferroelectrolyte. The interfacial evolution was investigated through EIS spectra at different cycle numbers at 1C in Figure S24 Supporting Information. During cycling, the Na_3_V_2_(PO_4_)_3_/PH‐5% N/HC cell exhibited a significant increase in interfacial impedance whereas the PH‐5% NC‐based full cell showed only minor impedance fluctuations, demonstrating the effectiveness of PH‐5% NC metaferroelectrolyte in stabilizing the electrode‐electrolyte interfaces.

## 3 Conclusion

In summary, we demonstrated flexible metaferroelectrolytes for highly efficient, stable, and compact SSB energy storage by introducing strongly ferroelectric‐coupled intrinsic ion conducting Na_2.99_Ba_0.005_OCl/CNNO^−^ nanosheets into the PVDF‐HFP matrix. Na_2.99_Ba_0.005_OCl acts as both an ionic conductor and an initiator to induce in situ chemical cross‐linking and spontaneous bridging of the PVDF‐HFP chains. The synergistic effect of good intrinsic elasticity and unique 3D ferroelectric network significantly enhances the conformal compatibility and stability of the electrolyte‐electrode interfaces in solid‐state sodium metal batteries, leading to a reduction in the interfacial resistance. Benefiting from strongly coupled interfacial ferroelectric engineering and the Na_2.99_Ba_0.005_OCl/CNNO^−^‐induced PVDF‐HFP ferroelectric β phase, the Na^+^ flux is regulated, effectively enhancing ion transport and inhibiting Na dendrite formation and growth at the interface. A systematic study shows that the assembled Na_3_V_2_(PO_4_)_3_/PH‐5% NC/Na batteries demonstrated excellent cycle stability and high Coulombic efficiency at room and even lower temperatures. Most importantly, the obtained metaferroelectrolytes exhibit superior fireproofing properties and energy densities for compact SSBs. This study provides a promising strategy for designing functional PVDF‐based metaferroelectrolytes for scalable and compact SSB energy storage, even to solid‐state irregular shaped batteries including roll‐to‐roll or meta‐structured fiber batteries. The conformal construction of an ultrathin multi‐functional metaferroelectrolyte results in cost‐effective, safe, stable, and compact SSBs with enhanced energy densities. Integrating strongly coupled ferroelectric ionics into solid‐state metal battery systems induces strong ferro‐electrochemical coupling. The flexible multi‐functional metaferroelectrolytes enable sapiential battery systems beyond traditional electrochemical energy storage devices.

## Conflict of Interest

The authors declare no conflict of interest.

## Supporting information



Supporting Information

## Data Availability

The data that support the findings of this study are available from the corresponding author upon reasonable request.
