# Supplementary material for: A Cross‐Linked Flexible Metaferroelectrolyte Regulated by 2D/2D Perovskite Heterostructures for High‐Performance Compact Solid‐State Sodium Batteries
Source: Adv Sci (Weinh). 2025 Jun 23;12(28):2416662. doi: 10.1002/advs.202416662 (PMC12302523; doi:10.1002/advs.202416662)
Supplement: Supplementary file 1 — Supporting Information [file ADVS-12-2416662-s001.docx]

Supporting Information

A Cross-Linked Flexible Metaferroelectrolyte Regulated by 2D/2D Perovskite Heterostructures for High-Performance Compact Solid-State Sodium Batteries

Yanan Huang, Zhuo Yang, Weicheng Zhou, Liequan Liu, Chuanbao Tu, Mingyang Tang, Haijiao Xie, Yu Lu, Xu Yan, Zhihao Ding, Xiaolong Li, Tiannan Yang, Alexander S. Sigov, Wei Huang, Lijun Gao and Cheng Huang*

Y.N. Huang, Z. Yang, W.C. Zhou, L.Q. Liu, C.B. Tu, H.J. Xie, Prof. L.J. Gao, Prof. C. Huang

Volta and DiPole Materials Labs, College of Energy

Soochow Institute for Energy and Materials InnovationS (SIEMIS)

Soochow Innovation Consortium for Intelligent Fibers and Wearable Technologies

Key Laboratory of Advanced Carbon Materials and Wearable Energy Technologies of Jiangsu Province

Key Laboratory of Core Technology of High Specific Energy Battery and Key Materials for Petroleum and Chemical Industry

Soochow University, 688 Moye Road, Suzhou 215006, P. R. China

E-mail: [chengh@suda.edu.cn](mailto:chengh@suda.edu.cn)

Y.N. Huang, Yu Lu, Xu Yan, Zhihao Ding, Prof. A.S. Sigov, Prof. C. Huang

International Joint Metacenter for Advanced Photonics and Electronics

Physics and Energy Department, School of Optical and Electronic Information

Suzhou City University, 1188 Wuzhong District, Suzhou 215006, P. R. China

E-mail: [chengh@szcu.edu.cn](mailto:chengh@suda.edu.cn)

Y.N. Huang, Z. Yang, W.C. Zhou, M.Y. Tang, Prof. C. Huang

High Density Materials Technology Center for Flexible Hybrid Electronics

Innovation Center for MIIT China Prosperity Green Industry Foundation &

Industry Development Research Institute

Xi’an Jiaotong University-Suzhou Institute of Electronic Functional Materials Technology

Suzhou Industrial Technology Research Institute, Suzhou 215151, P. R. China

Prof. T.N. Yang

Interdisciplinary Research Center, School of Mechanical Engineering

Shanghai Jiao Tong University, 800 Dongchuan Road, Shanghai 200240, P.R. China

Prof. A.S. Sigov, Prof. C. Huang, Prof. W. Huang

School of Flexible Electronics & State Key Laboratory of Optoelectronic Materials and Technologies

Sun Yat-sen University, 66 Gongchang Road, Guangming District, Shenzhen 518107, P.R. China

Prof. A.S. Sigov

Nanoelectronics Department, MIREA-Russian Technological University, 78 Vernadsky Ave., Moscow 119454, Russia

Dr. X.L. Li

Shanghai Synchrotron Radiation Facility, Shanghai Advanced Research Institute

Shanghai Institute of Applied Physics, Chinese Academy of Sciences

Shanghai 201204, P. R. China

Prof. C. Huang

Pacific Northwest National Laboratory, Richland, Washington 99352, USA

Y.N. Huang, Prof. C. Huang, Prof. W. Huang

Institute of Advanced Materials and Institute of Membrane Science and Technology

Jiangsu National Synergistic Innovation Center for Advanced Materials

State Key Laboratory of Flexible Electronics, Suzhou Laboratory and Nanjing Tech University, Nanjing 211816, P. R. China

**Experimental Section**

*Materials:* All chemicals were used without further purification. Nb_2_O_5_ (99%), CaCO_3_ (99%), K_2_CO_3_ (99%), Na_2_CO_3_ (99%), and *N, N*-dimethylformamide (DMF, 99.8%) were obtained from Aladdin. NaOH (99%), NaCl (99%), Ba(OH)_2_·8H_2_O (98%), and NaTFSI (98%) were obtained from Macklin. Tetrabutylammonium hydroxide (TBAOH, 10% in H_2_O) and poly(vinylidene fluoride-co-hexafluoropropylene) (PVDF-HFP, Mw ~ 400,000 g mol^-1^) were purchased from Aladdin.

*Preparation of* *Ca_2_Na_2_Nb_5_O_16_*^-^ *Nanosheets:* Ca_2_Na_2_Nb_5_O_16_^-^ (CNNO^-^) nanosheets were prepared by liquid-phase exfoliation according to previously reported procedures.^[1,2]^ The starting materials, KCa_2_Nb_3_O_10_ and NaNbO_3_, were prepared via solid-state synthesis. The KCa_2_Nb_3_O_10_ powder was obtained by calcinating a mixture of K_2_CO_3_, CaCO_3_, and Nb_2_O_5_ (K/Ca/Nb = 1.1/2/3 in molar ratio) at 1200 °C for 12 h in air atmosphere, while NaNbO_3_ powder was prepared by mixing Na_2_CO_3_ and Nb_2_O_5_ (Na/Nb = 1 in molar ratio) and calcinated at 1200 °C for 12 h in air atmosphere. Afterward, a mixture of KCa_2_Nb_3_O_10_ and NaNbO_3_ (Na/Ca = 1 in molar ratio) was heated at 1300 °C for 24 h to synthesize the bulk KCa_2_Na_2_Nb_5_O_16_ material and then converted into the protonic form HCa_2_Na_2_Nb_5_O_16_ in 5 M HNO_3_ solution for 72 h. Finally, the washed and filtered resulting powder (1.2 g) was dispersed in a 400 mL aqueous solution of tetrabutylammonium hydroxide (TBAOH) for 7 days. The colloidal solution was centrifuged at 10000 rpm for 30 min to produce CNNO^-^ nanosheets.

*Preparation of Na_2.99_Ba_0.005_OCl/Ca_2_Na_2_Nb_5_O_16_*^-^ *Heterostructures:* Typically, the Na^+^ amorphous-oxide (glass) electrolytes were synthesized by weighing NaCl, NaOH, and Ba(OH)_2_·8H_2_O with a stoichiometric ratio and adding 30 mL of deionized water. The resulting aqueous solutions were mixed and stirred for 2 h at 80 °C until all the reagents were dissolved. Subsequently, a certain amount of CNNO^-^ nanosheets were added to the uniformly mixed solution and continuously stirred for 2 h. The obtained solution was sealed in a 50 mL Teflon reactor and heated at 240 °C for at least 4 days. Na_2.99_Ba_0.005_OCl/CNNO^-^ precipitated in the Teflon reactor as a white solid when the water had completely evaporated. The obtained materials were placed in an Ar-filled glove box. Na_2.99_Ba_0.005_OCl was prepared using the same procedure but without the addition of CNNO^-^ nanosheets.

*Preparation of PVDF-HFP Metaferroelectrolyte Membranes:* The PVDF-HFP particles and NaTFSI powder were dried at 80 °C for 12 h. Firstly, 0.4 g PVDF-HFP particles and 0.1 g NaTFSI were dissolved in DMF and stirred for 1 h at 60 °C until all the reagents were dissolved. Afterward, 5 wt % Na_2.99_Ba_0.005_OCl/CNNO^-^ powders or Na_2.99_Ba_0.005_OCl (compared to the weight of PVDF-HFP) were added into the solution to induce chemical cross-linking followed by stirring at 60 °C for 4 h. Then, the solution was cast into a glass mold under vacuum at 80 °C for 12 h to evaporate the solvent. The resulting PVDF-HFP metaferroelectrolyte membranes were transferred to a glove box for further use. The same procedure was followed to prepare the PVDF-HFP metaferroelectrolyte membranes by adding different amounts of Na_2.99_Ba_0.005_OCl/CNNO^-^ powders.

*Cathode Preparation:* Na_3_V_2_(PO_4_)_3_ powder, acetylene black, and polyvinylidene fluoride binder (8:1:1 mass ratio) were dispersed in *N*-methyl pyrrolidone to form a uniform slurry. After that, the slurry was coated on Al foil and dried at 80 °C overnight under vacuum. The average mass loading of Na_3_V_2_(PO_4_)_3_ electrode material was between 2 and 3 mg cm^-2^. Finally, the Na_3_V_2_(PO_4_)_3_ cathode (theoretical capacity of ≈117 mA h g^−1^) was punched into 14 mm disks for use.

*Materials Characterizations:* XRD (D8-ADVANCE, Bruker) was used to analyze the crystal structure and phase information of the samples using Cu Kα radiation (λ = 1.5418 Å). The sample morphologies were revealed by cold-field emission SEM (Hitachi SU8010) with an accelerating voltage of 5 kV and TEM (JEOL JEM-2100F) with an accelerating voltage of 80 kV. Quantitative element mapping was performed using energy-dispersive X-ray spectroscopy (EDX). The surface topographies and thicknesses of the samples were determined using a Bruker Dimension Icon atomic force microscope (AFM). The XPS spectra were obtained from a Thermo Scientific ESCALAB250 X-ray photoelectron spectrometer to determine the surface chemical bonding states. Fourier transform infrared spectrometer (FTIR, Bruker) was hired to evaluate functional groups or bonding properties in the polymer in the range of 2500-600 cm^-1^. The 3D images of the samples were captured by XCT (XRADIA 620 VERSA, ZEISS) with 1μm resolution. 2D GIWAXS tests were conducted to examine the orientation and arrangement of the nanosheets within the membranes using the BL02U2 surface diffraction beamline at the Shanghai Synchrotron Radiation Facility. The X-ray wavelengths were 1.24 Å. AFM-IR measurement was conducted on an Infrared Scanning Near-field Optical Microscope (Bruker NanoIR3-s) to obtain the morphological and chemical images in tapping mode with a spectra range of 870-1470 cm^-1^ and a resolution of 2 μm. The polarization-electric field (P-E) loop was collected by TF analyzer ferroelectric test system (aix ACCT 2000). The CNNO^-^ powder was pressed into a round piece with a thickness approximately 500 μm, and gold electrodes were deposited on both its top and bottom surfaces to test. DSC (822e) was performed to analyze changes in the crystallinity of the polymers. Tensile-strain curves were measured using a tensile machine (HY-0580) at a stretching speed of 10 mm min^-1^. For visualization of the dendrtie growth, 12.0 mg 4-(1,2,2-triphenylvinyl) phenol (TPEOH) was dissolved in 2 mL anhydrous ether, and then the solution was dropped on the sodium surface. Fluorescence microscopy was performed to analyze the sodium surface after the reaction and evaporation.

*Electrochemical Measurements:* All electrochemical tests were conducted using a LAND CT2001A battery test system and an electrochemical workstation (CHI660E). Unless otherwise specified, all electrochemical tests were performed at room temperature. The PVDF-based electrolytes were punched into circular sheets with diameters of 18 mm. The ionic conductivity (*σ*) of the PVDF-based electrolytes was calculated by EIS between two stainless steel (SS) blocking electrodes with a frequency range from 1 MHz to 0.1 Hz between 5 and 55 °C. The *σ* values can be calculated by the following Equation (1),

*σ =* $\frac{L}{RS}$ (1)

where L (cm) is the thickness of the PVDF-based electrolyte membrane, R (Ω) is the bulk resistance of the electrolyte, and S (cm^2^) is the contact area of the electrode and electrolyte.

The Na^+^ transference number (t_Na+_) of the PVDF-based electrolyte membrane was determined by direct current (DC) polarization (with a DC voltage of 10 mV) combined with alternating-current (AC) EIS (from 1 MHz to 0.1 Hz) using Na/electrolyte/Na symmetric cells. t_Na+_ values were obtained using Equation (2).

t_Na+_ =$\frac{I_{s}(\Delta V-I_{0}R_{0})}{I_{0}(\Delta V-I_{s}R_{s})}$ (2)

in which the ΔV refers to the amplitude of applied polarization voltage (10 mV), R_0_ and R_s_ are the interfacial impendence before and after polarization according to the EIS, and I_0_ and I_s_ represent initial and steady-state current. The electrochemical stability window of PVDF-based electrolytes was investigated by linear sweep voltammograms (LSV) using Na/electrolytes/SS from 1 to 6 V at a scan rate of 1 mV s^-1^. Moreover, the stripping/plating behavior of PVDF-based electrolytes was carried out on Na /electrolytes/ Na symmetric cells at a current density of 0.2 and 1 mA cm^-2^. The Na_3_V_2_(PO_4_)_3_||Na batteries were assembled in coin-type (CR2025) half-cells to evaluate the electrochemical properties of the electrolytes. Galvanostatic charge-discharge characterization was tested in the 2.6 to 4.0 V voltage range. CV curves were collected in 2.6-4.0 V at a scan rate of 0.2 mV s^-1^. The Na_3_V_2_(PO_4_)_3_||HC full cells were assembled in coin-type (CR2025) cells to evaluate the electrochemical properties of the electrolytes, using hard carbon (HC) as the anode, Na_3_V_2_(PO_4_)_3_ as the cathode (4 mg cm^-2^ loading), and PH-5% NC electrolyte, with a controlled N/P ratio of 1.1. Galvanostatic charge-discharge characterization was tested in the 1.5 to 3.9 V voltage range under 25 °C.

*Energy Density Estimation:* The energy density is estimated based on pouch cells, and the following are the relevant formulas and specific parameters for estimation:

Volumetric energy density: $\frac{Wh}{L}$ =$\frac{Ccathode*Vcathode}{L}$ (3)

Gravimetric energy density: $\frac{Wh}{Kg}$ =$\frac{Ccathode*Vcathode}{Kg}$ (4)

where *C* represents capacity, *V* represents average working voltage of electrode material. By adopting the Na_3_V_2_(PO_4_)_3_ as the cathode material, the *C_cathode_* is 117 mAh g^-1^, *V_cathode_* is 3.4 V, the thickness of PH-5% NC is ~50 μm and the weight is ~9.5 mg cm^-2^, and its volumetric energy density is calculated at ~ 306.9 Wh L^-1^.

*Ferroelectric Potential/Electric Field Distribution Simulation and Ionic Coupling DFT Calculation:* The μ-PRO® software was used to simulate the intrinsic ferroelectric potential distribution and internal electric field from the Ca_2_Na_2_Nb_5_O_16_^-^ nanosheet. The electrostatic equilibrium Equation (5) was solved as follows.

$\nabla\cdot\left( \kappa_{0}\kappa^{b}E+P \right)=\rho_{f}.$ (5)

Here, $\kappa_{0}$ and $\kappa^{b}$ are the vacuum permittivity and background dielectric constant, respectively. $E\left( x \right)$ is the electric field to be solved, which is related to the electric potential field $\Phi\left( x \right)$ by $E=-\nabla\Phi$, with $x$ being the position vector. $P\left( x \right)$ is the ferroelectric polarization field, and $\rho_{f}=0$ is the free charge density. The background dielectric constant of the ferroelectric is taken as $\kappa^{b}=40$ following the common value of various ferroelectric oxides,^[3]^ and the magnitude of the polarization of is taken as *P* = 0.20 C m^-2^, where the direction of polarization is parallel to the (111) plane compared to the (001) or (100) plane. A simplified model of 3-dimensional CNNO^-^ nanosheet with a total size of 100×100×3 nm^3^ is considered, which is discretized into 100×100×6 grid cells for the numerical solution. An open-circuit boundary condition is employed for solving the electrostatic equilibrium Equation (5). A Fourier spectral iteration perturbation method^[4]^ is employed for the numerical solution. DFT calculations of ion coupling and transport^[5,6]^ were performed using the Vienna *ab initio* simulation package (VASP) relying on plane-wave basis sets using the projector augmented-wave method.^[7,8]^ The exchange-correlation potential was handled using a generalized gradient approximation with Perdew (Burke) Ernzerhof parameterization.^[9]^ We implemented a van der Waals correction using Grimme's DFT-D3 model to enhance our calculations, and an energy cutoff of 520 eV was set. For Brillouin-zone integration, we sampled with a Γ-centered Monkhorst-Pack mesh of 2 × 2 × 1 tailored specifically for the interface. When the maximum force on each atom was less than 0.01 eV/Å, the structures were fully relaxed with the energy convergent standard of 10^-5^ eV, reaching the minimum energy principle during the structure optimization process. We employed the climbing-image nudged elastic band (CI-NEB) approach^[10]^ to calculate energy barriers. For the simulation of the external EF (12.44 mV nm^-1^), the method proposed by Neugebauer and Scheffler was used in the VASP package,^[11]^ where an artificial dipole sheet was introduced in the center of the vacuum space.

To explore the binding behavior of Na^+^ using different models, we calculated the adsorption energies by the following Equation (6),

E_ad_ = E_total_ - (E_model_ + E_Na_) (6)

where E_model_, E_Na_, and E_total_ denote the total energies of the pristine model (including the slab and interface models), a single Na atom (calculated from bulk Na), and a structural model (including the slab and interface models) with an adsorbed Na atom, respectively.

*Statistical Analysis:* Load-displacement curves were obtained from Nanoindenter (G200) with the compression velocity of 10 nm s^-1^ to assess electrolyte membrane flexibility. The test loading force was 2 mN. To ensure data accuracy, measurements were conducted at five distinct locations on each sample.


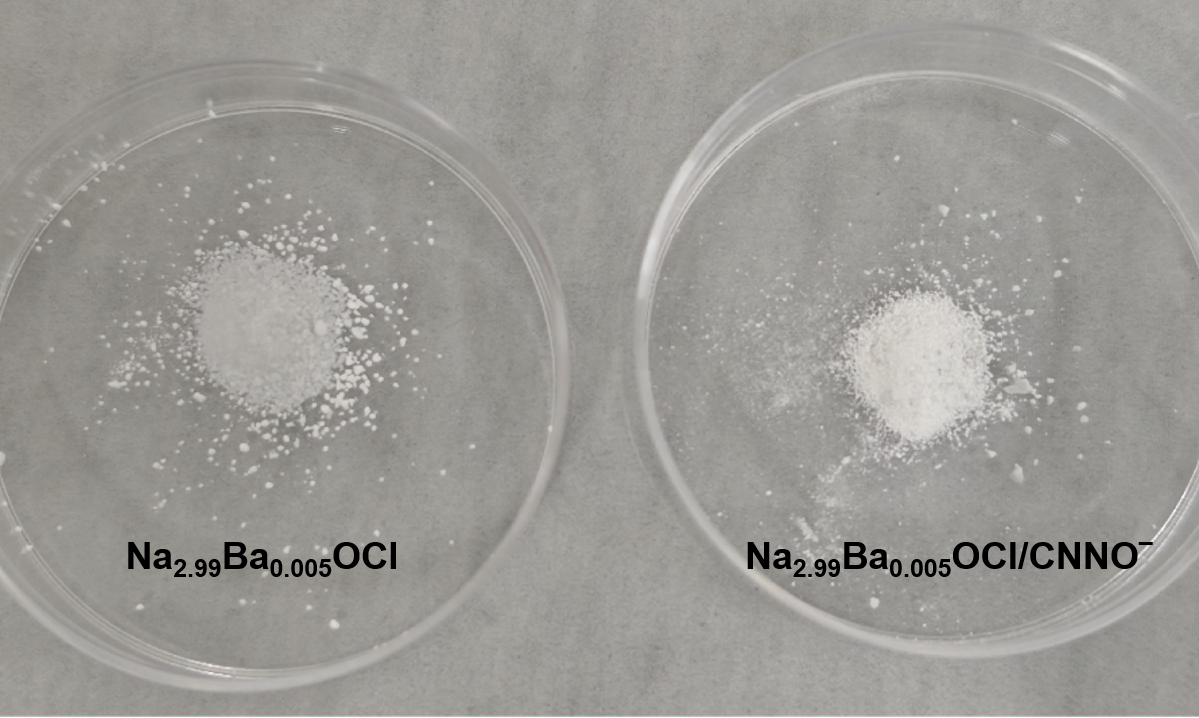


**Figure S1.** The visual comparison of air stability between Na_2.99_Ba_0.005_OCl/CNNO^-^ and Na_2.99_Ba_0.005_OCl sample.


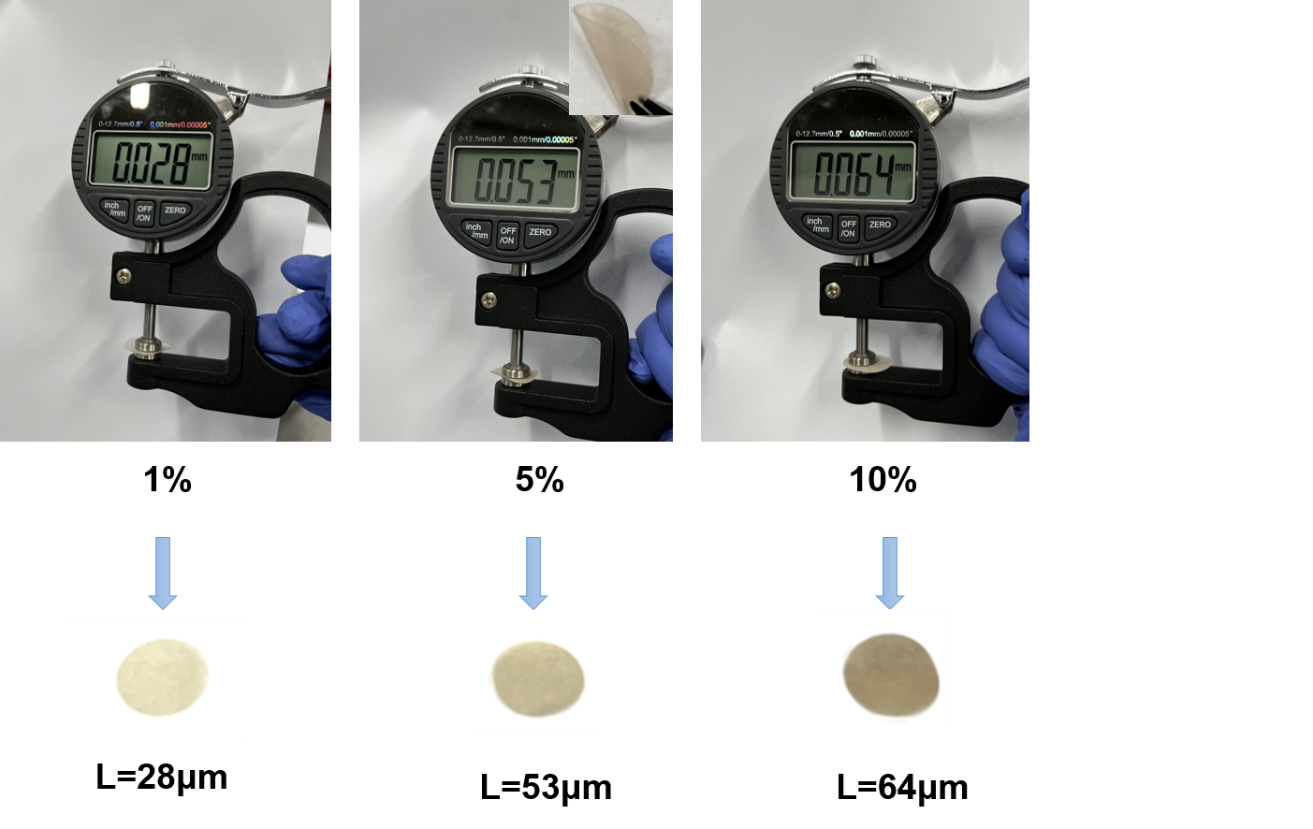

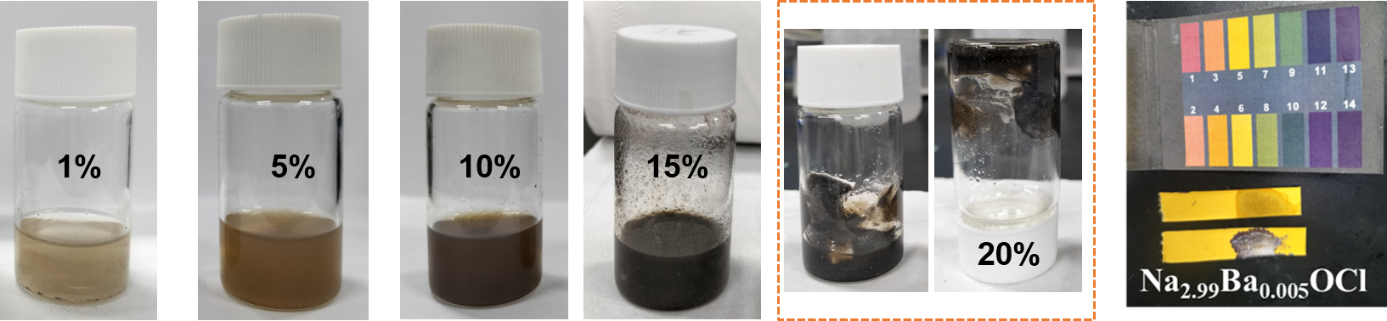


**Figure S2.** The digital photos of the samples for further details corresponding to the cross-linking phenomenon with different Na_2.99_Ba_0.005_OCl/CNNO^-^ content.


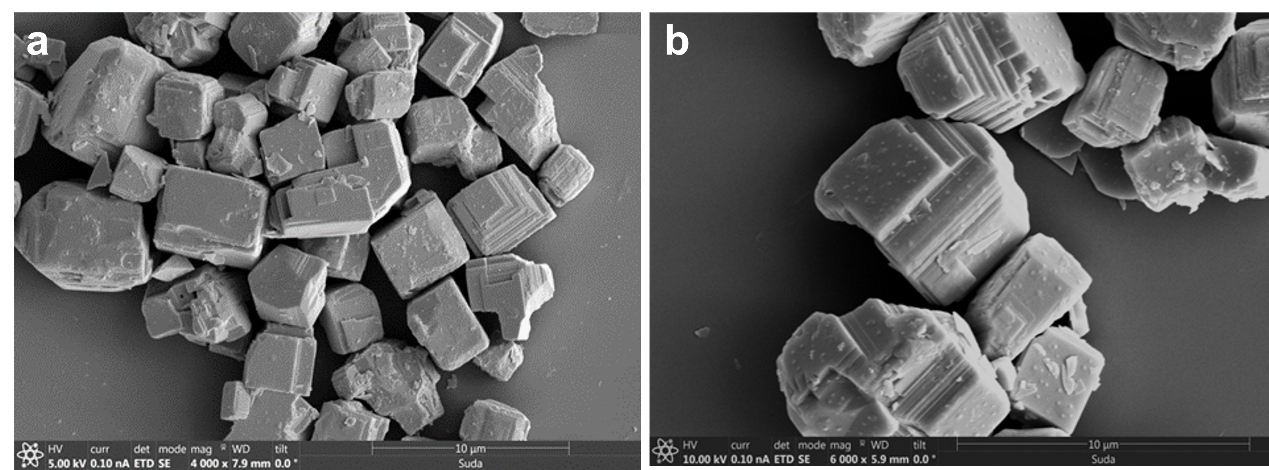


**Figure S3.** SEM images of KCa_2_Na_2_Nb_5_O_16_ before exfoliation.


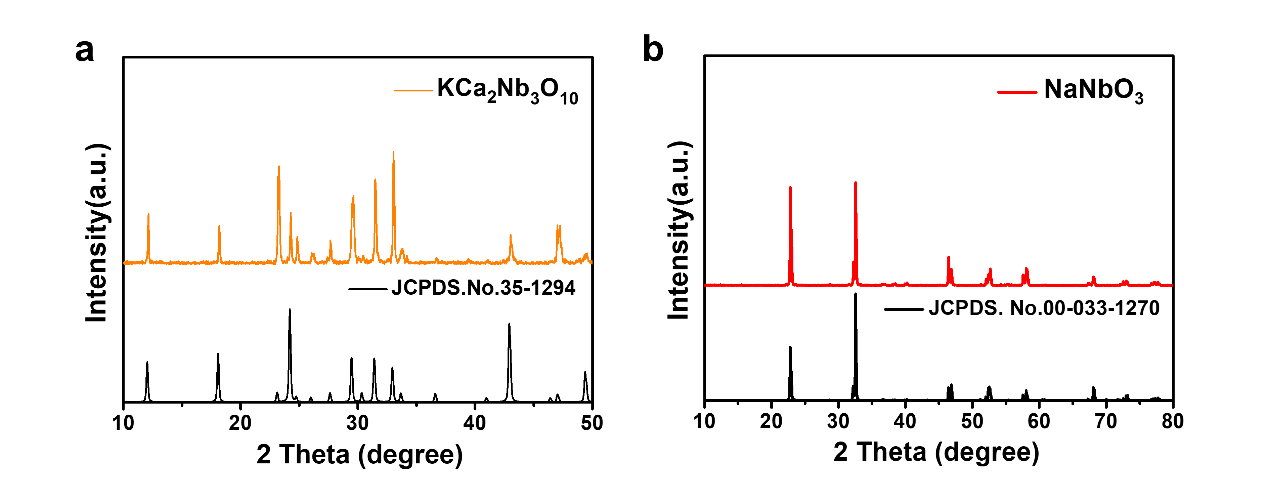


**Figure S4.** XRD patterns of a) KCa_2_Nb_3_O_10_ and b) NaNbO_3_.


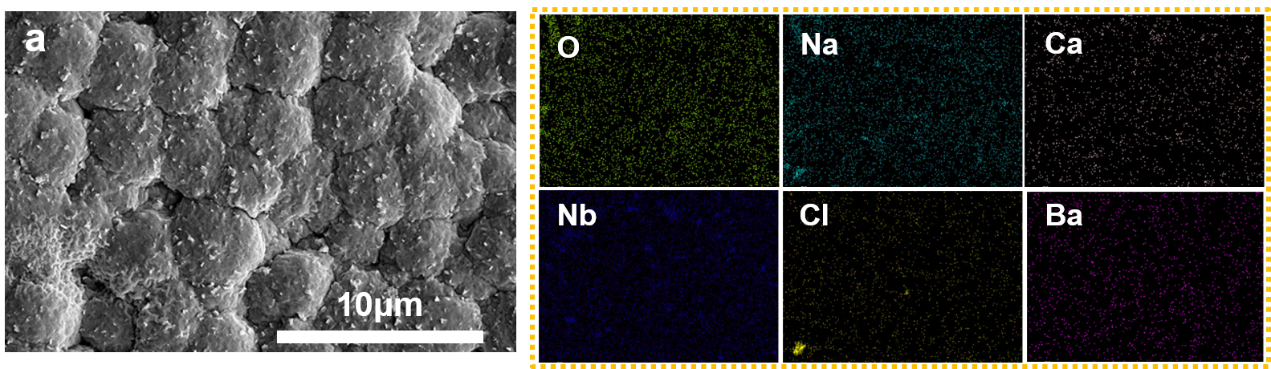


**Figure S5.** SEM image of top-view of PH-5% NC metaferroelectrolyte and corresponding EDS mappings.


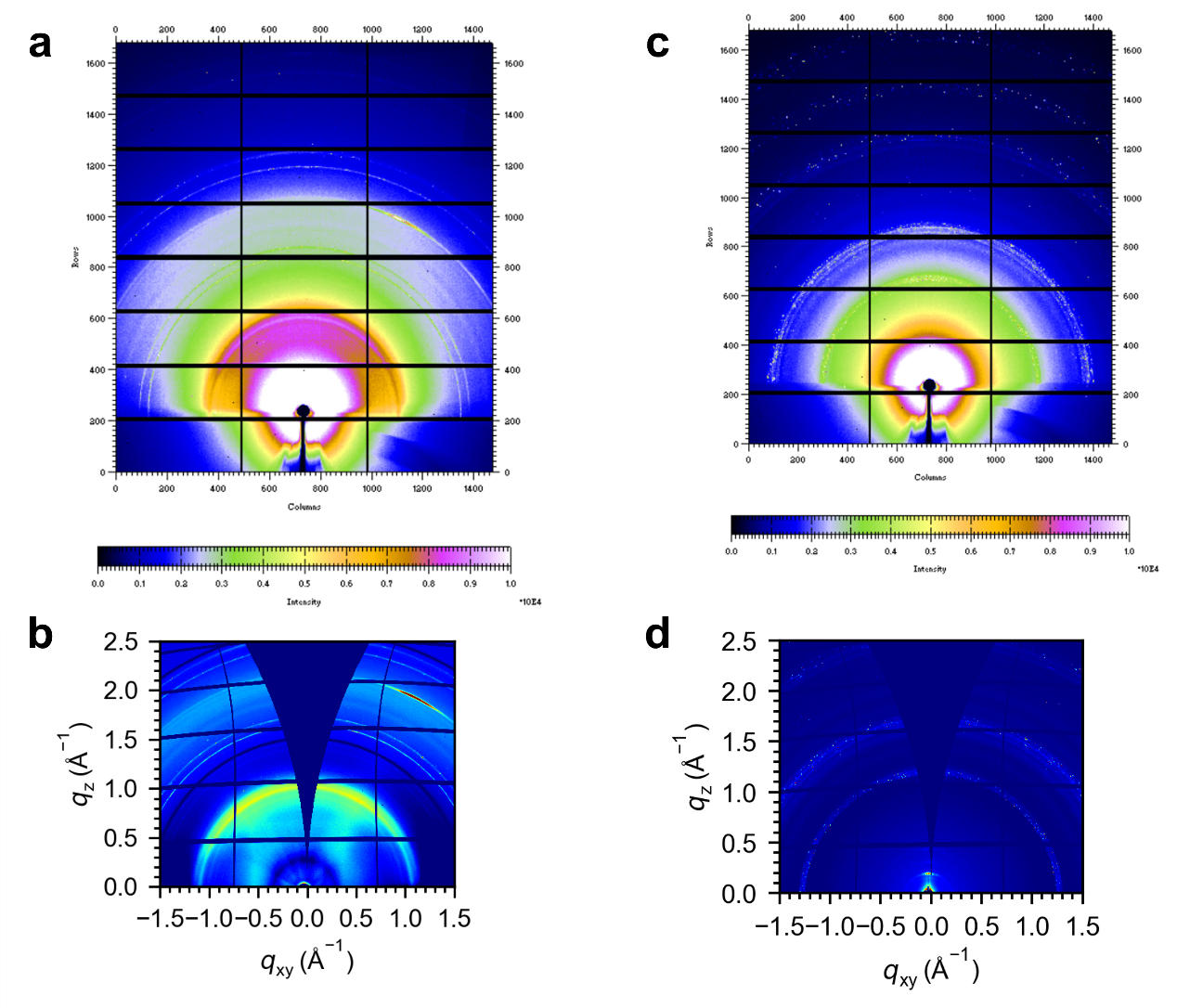


**Figure S6.** The pristine 2D-GIWAXS images of a) PH-5% NC metaferroelectrolyte and c) Na_2.99_Ba_0.005_OCl/CNNO^-^ heterostructure powder. Fixed images of prepared b) PH-5% NC metaferroelectrolyte and d) Na_2.99_Ba_0.005_OCl/CNNO^-^ heterostructure powder.


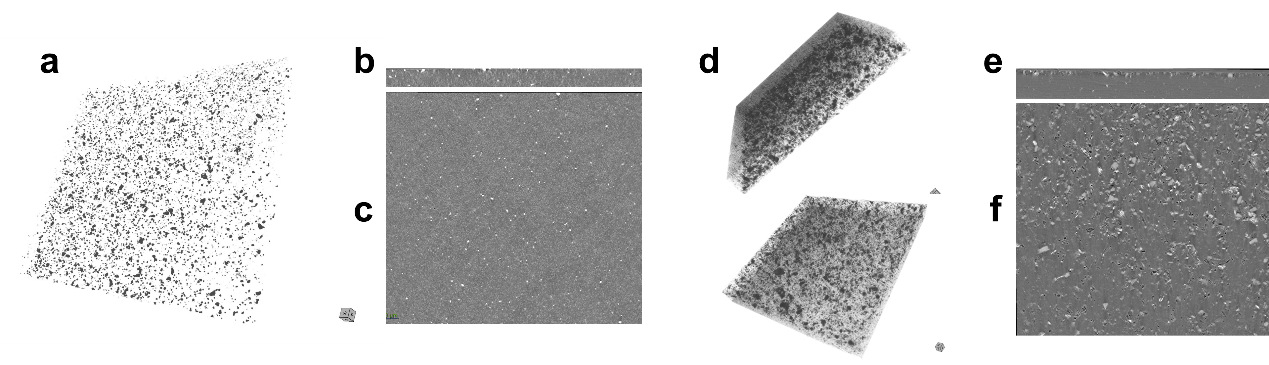


**Figure S7.** 3D reconstruction collected with XCT of a) PH-5% NC metaferroelectrolyte and d) PH-15% NC electrolyte. Cross-sectional and Surface XCT images of b, c) PH-5% NC metaferroelectrolyte and e, f) PH-15% NC electrolyte.


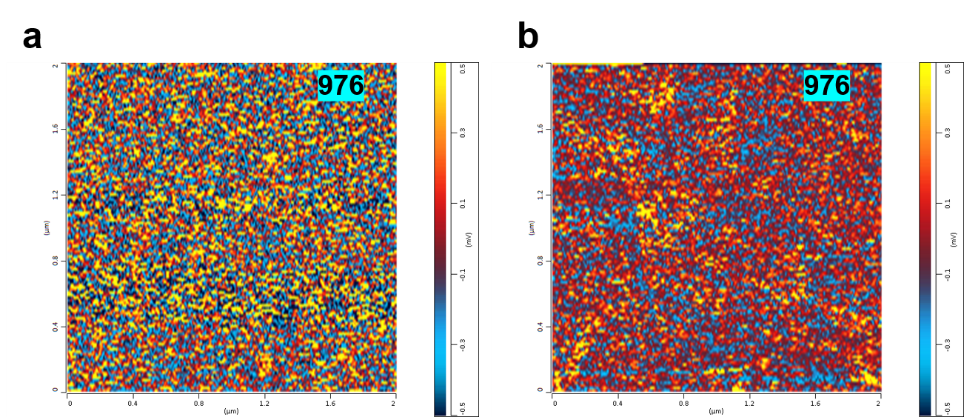


**Figure S8.** AFM-IR chemical mapping at 976 cm^-1^ of the a) PVDF-HFP and b) PH-5% NC.


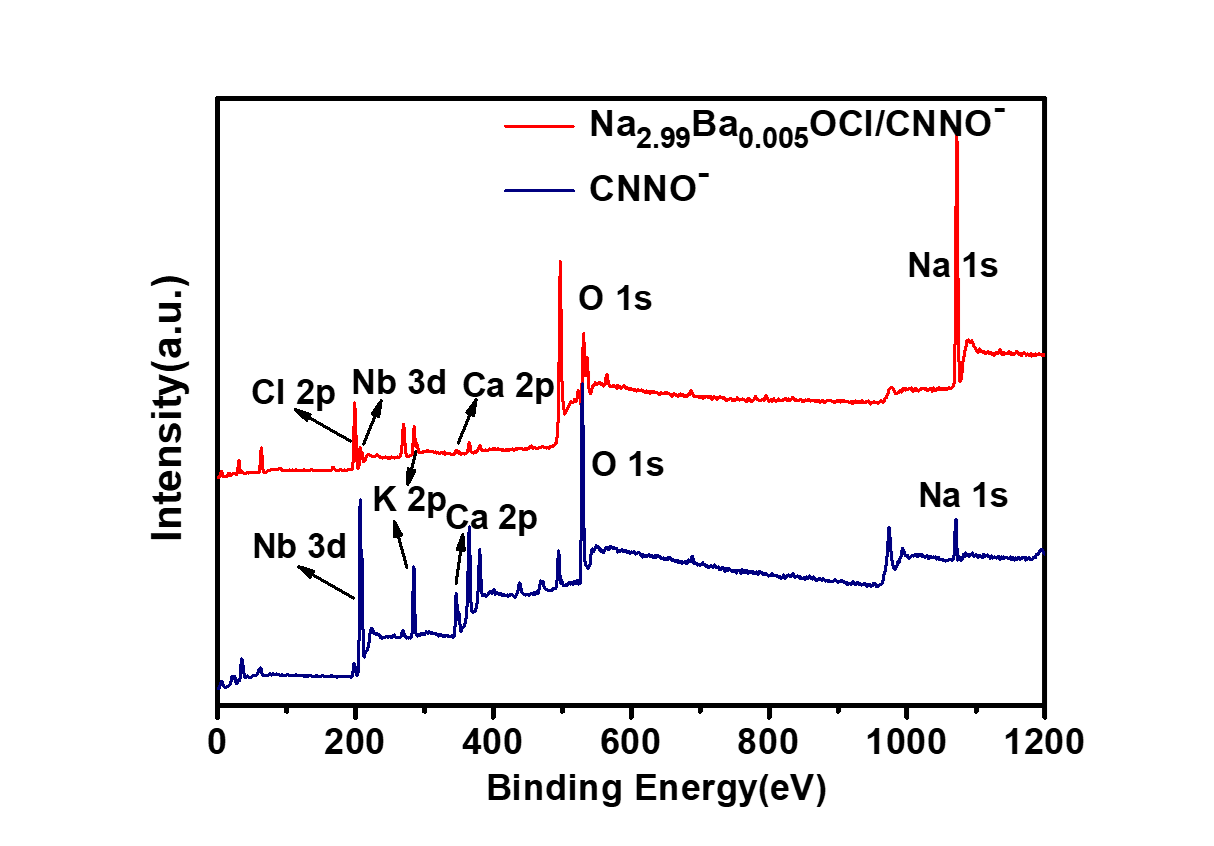


**Figure S9.** Full XPS survey spectrum of Na_2.99_Ba_0.005_OCl/CNNO^-^ and CNNO^-^.


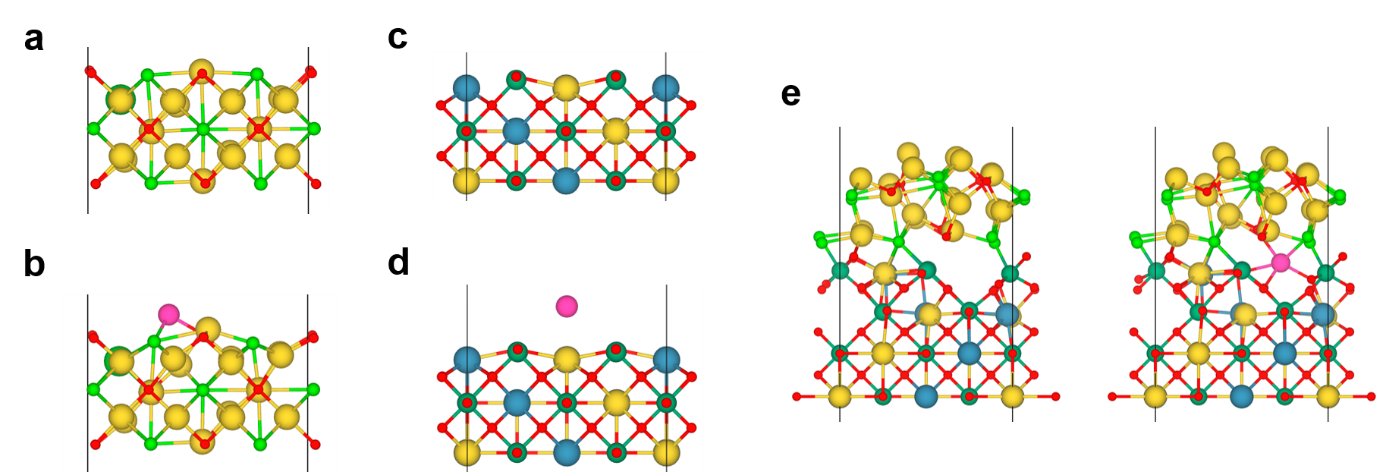


**Figure S10.** Side view of adsorbed sodium ion structure for a, b) Na_2.99_Ba_0.005_OCl; c, d) CNNO^-^ and e) Na_2.99_Ba_0.005_OCl/CNNO^-^. Spheres in models: yellow is Na, dark green is Nb, fluorescent green is Cl, blue is Ca, and red is O.


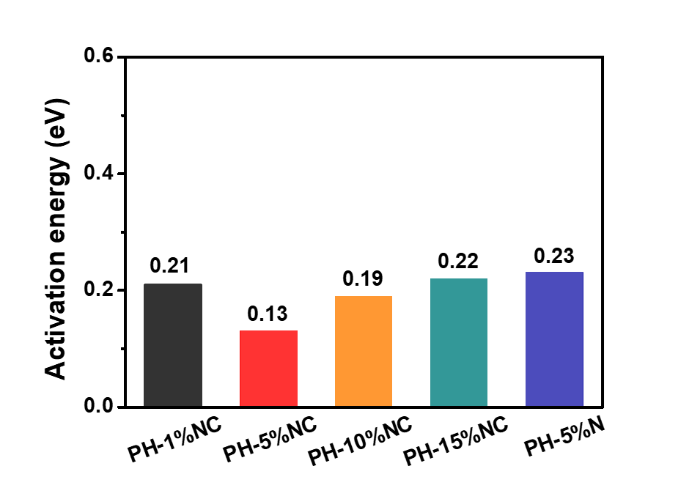


**Figure S11.** The activation energy required for ion transport of the PVDF-based electrolytes at 25°C.


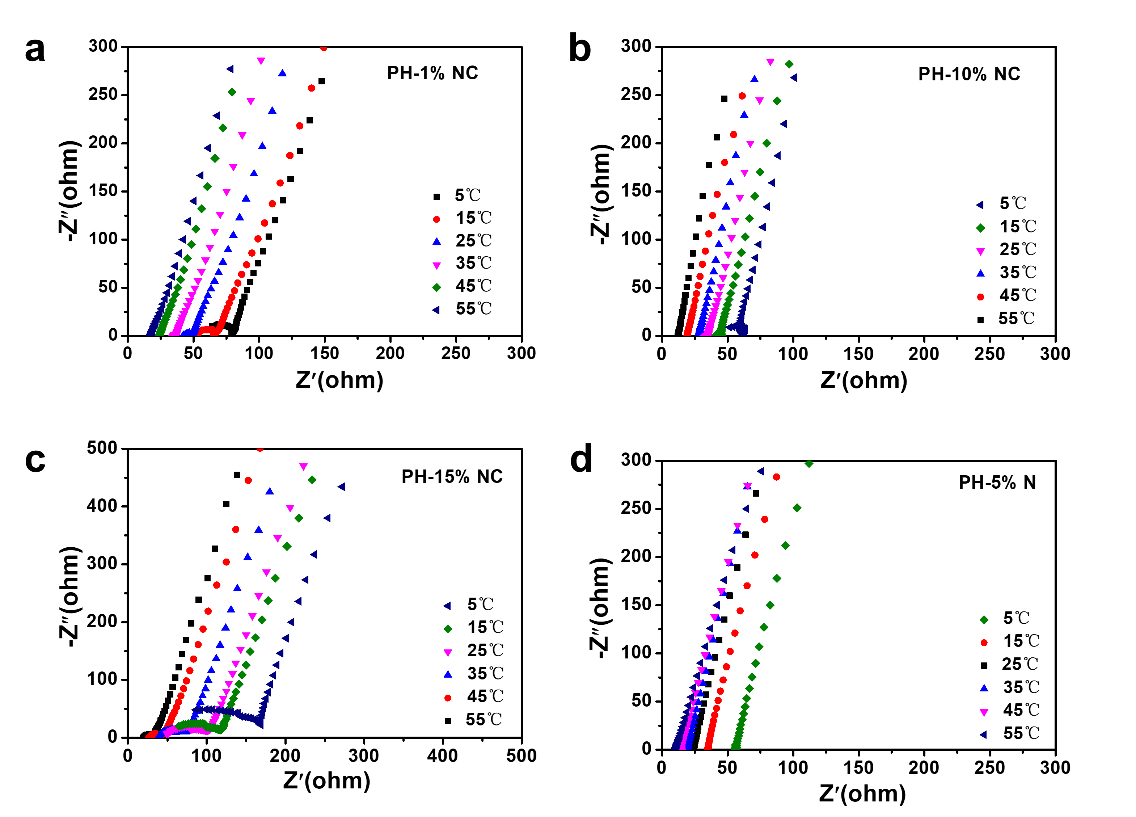


**Figure S12.** The EIS spectra of PVDF-based electrolytes with blocking electrode from 5°C to 55°C.


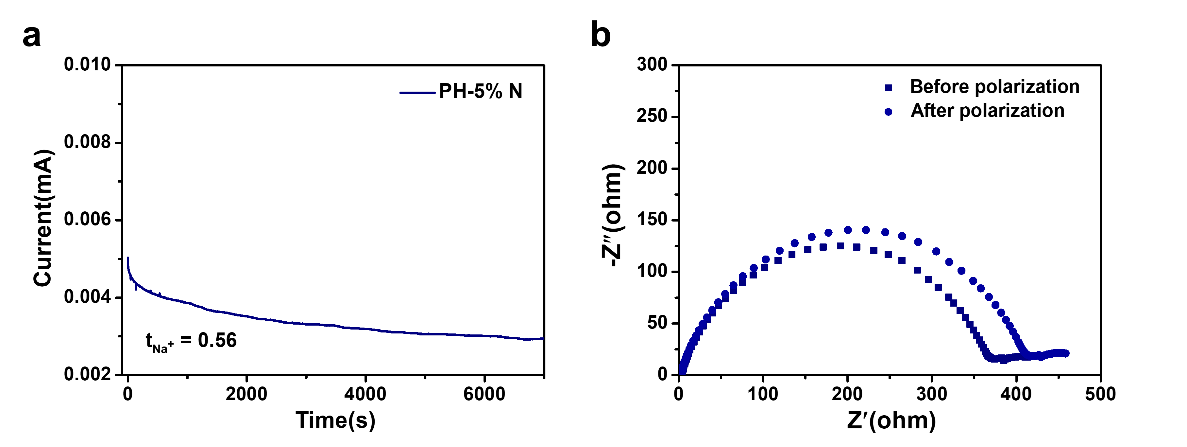


**Figure S13.** a) Current-time curve of the PH-5% N electrolyte and b) corresponding EIS spectra of Na/ PH-5% N/Na cell.


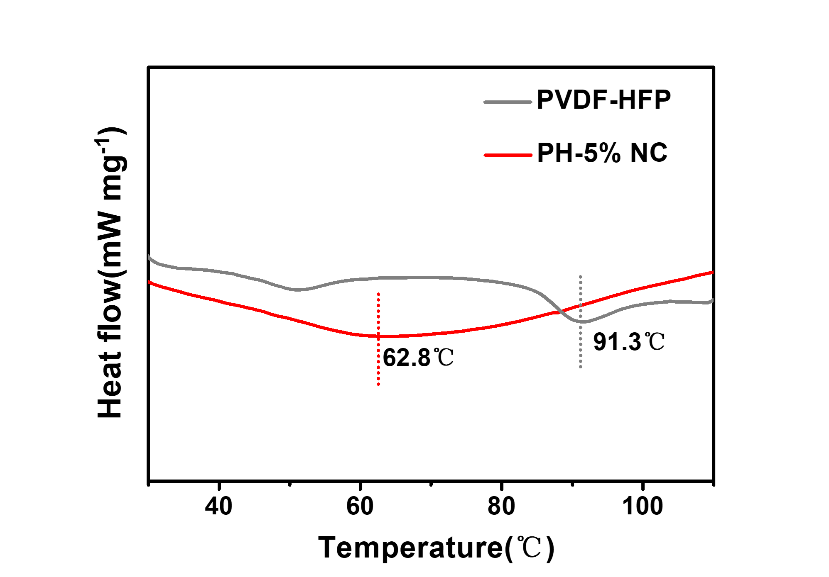


**Figure S14.** The DSC curves of PVDF-HFP and PH-5% NC metaferroelectrolyte.


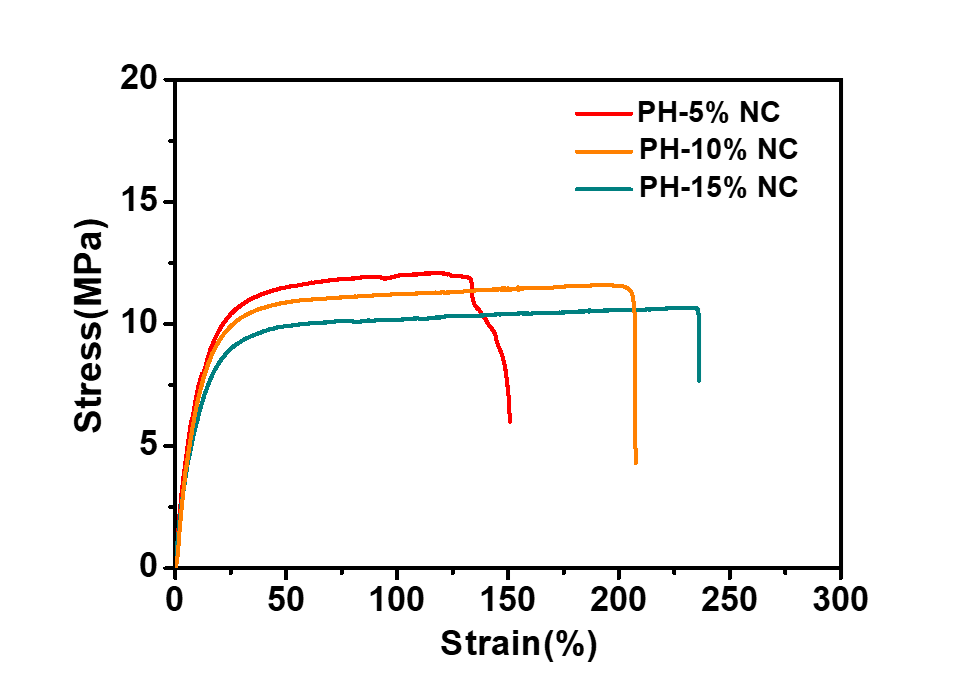


**Figure S15.** Stress-strain curves of PH-x% NC metaferroelectrolytes.


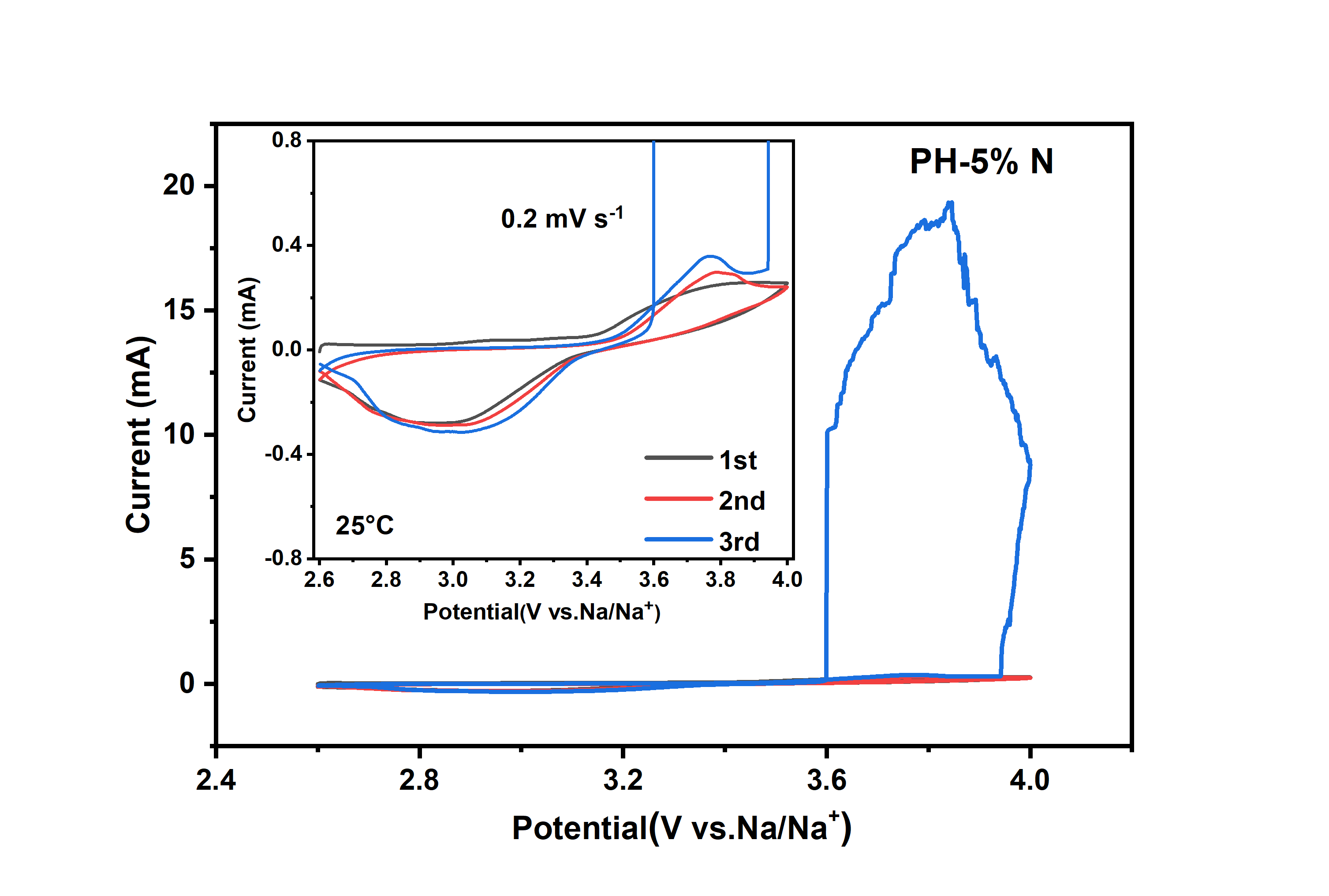


**Figure S16.** Cyclic voltammogram curves of Na_3_V_2_(PO_4_)_3_/PH-5% N/Na solid-state battery at 25 °C.


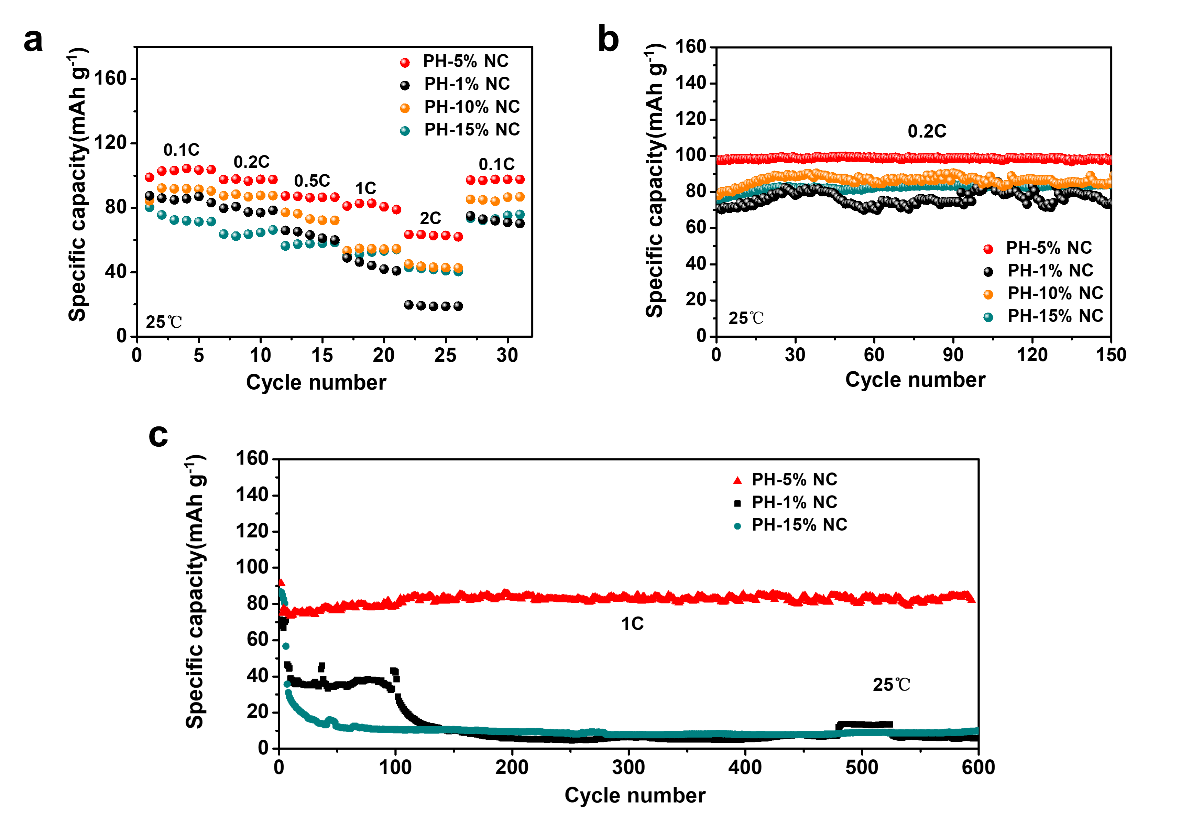


**Figure S17.** a) The rate performances of Na_3_V_2_(PO_4_)_3_/PH-x% NC/Na cell at 25 °C. b) Cycle performances of Na_3_V_2_(PO_4_)_3_/PH-x% NC/Na cells at 0.2C under 25 °C. c) Long-term cycling stability of Na_3_V_2_(PO_4_)_3_/PH-x% NC/Na cells at 1C under 25 °C.


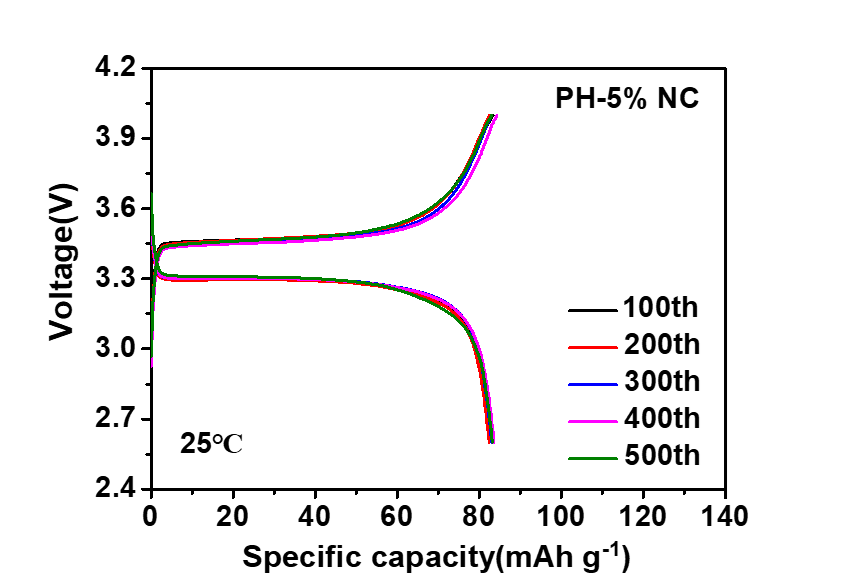


**Figure S18.** Charge-discharge curves of Na_3_V_2_(PO_4_)_3_/PH-5% NC/Na cell after different cycles at 1C under 25 °C.


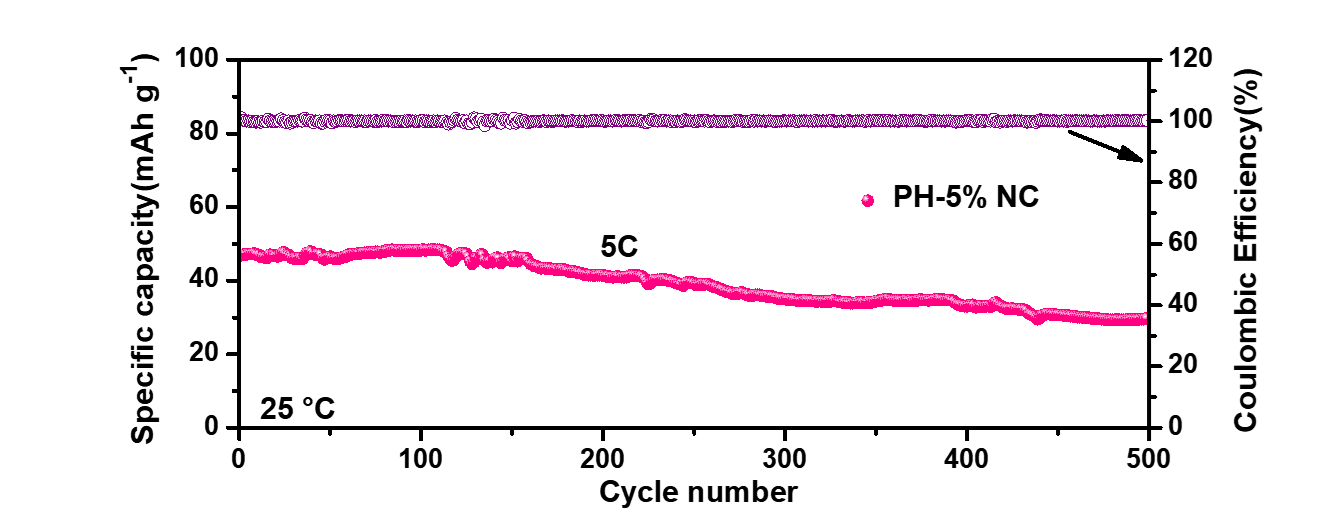


**Figure S19.** The long-term cycling stability of Na_3_V_2_(PO_4_)_3_/PH-5% NC/Na cells at 5C under 25 °C.


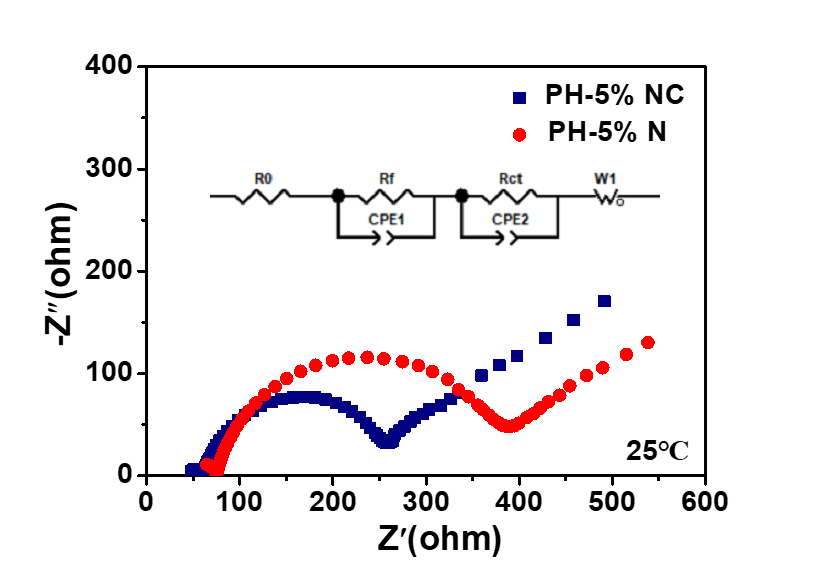


**Figure S20.** The EIS spectra of Na_3_V_2_(PO_4_)_3_//Na cells at 25 °C.


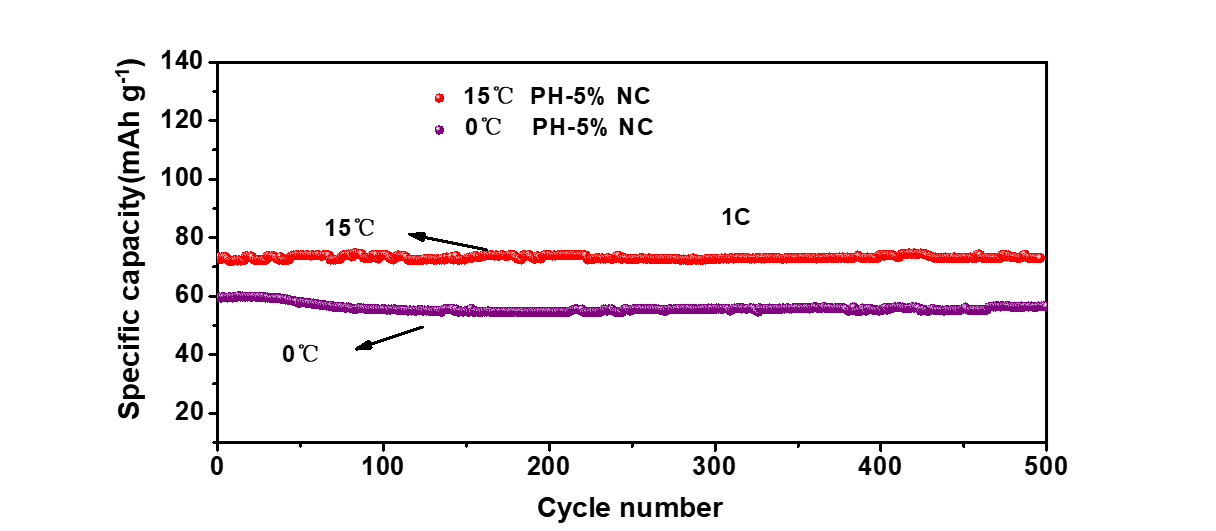


**Figure S21.** The long-term cycling stability of Na_3_V_2_(PO_4_)_3_/PH-5% NC/Na cells at 1C under 15 °C and 0 °C.


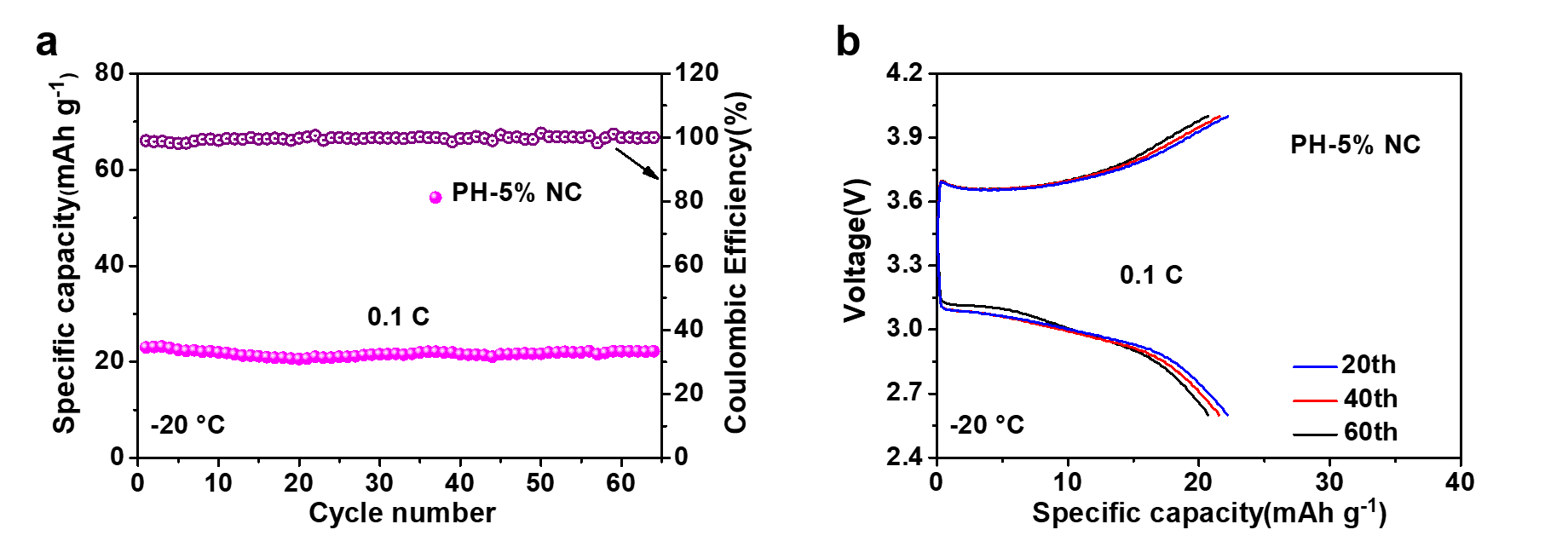


**Figure S22.** The cycling stability and charge-discharge curves of Na_3_V_2_(PO_4_)_3_/PH-5% NC/Na cells at 0.1C under -20 °C.


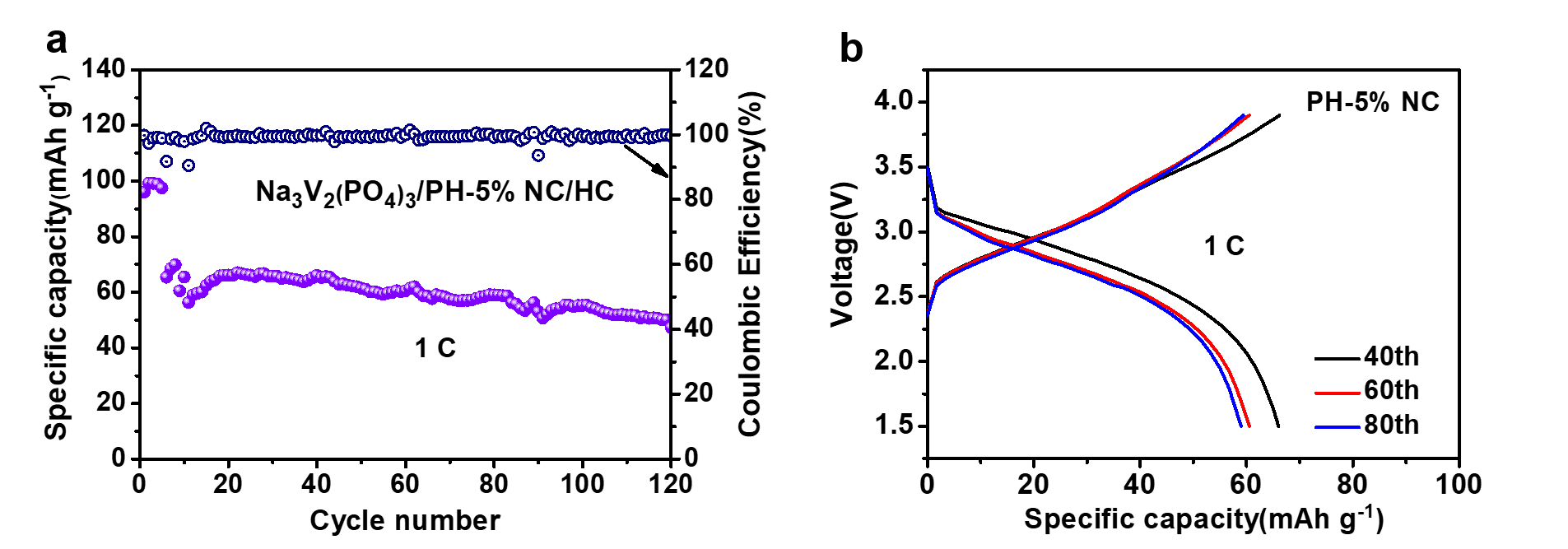


**Figure S23.** The cycling stability and charge-discharge curves of Na_3_V_2_(PO_4_)_3_/PH-5% NC/HC cells at 1C under 25 °C.


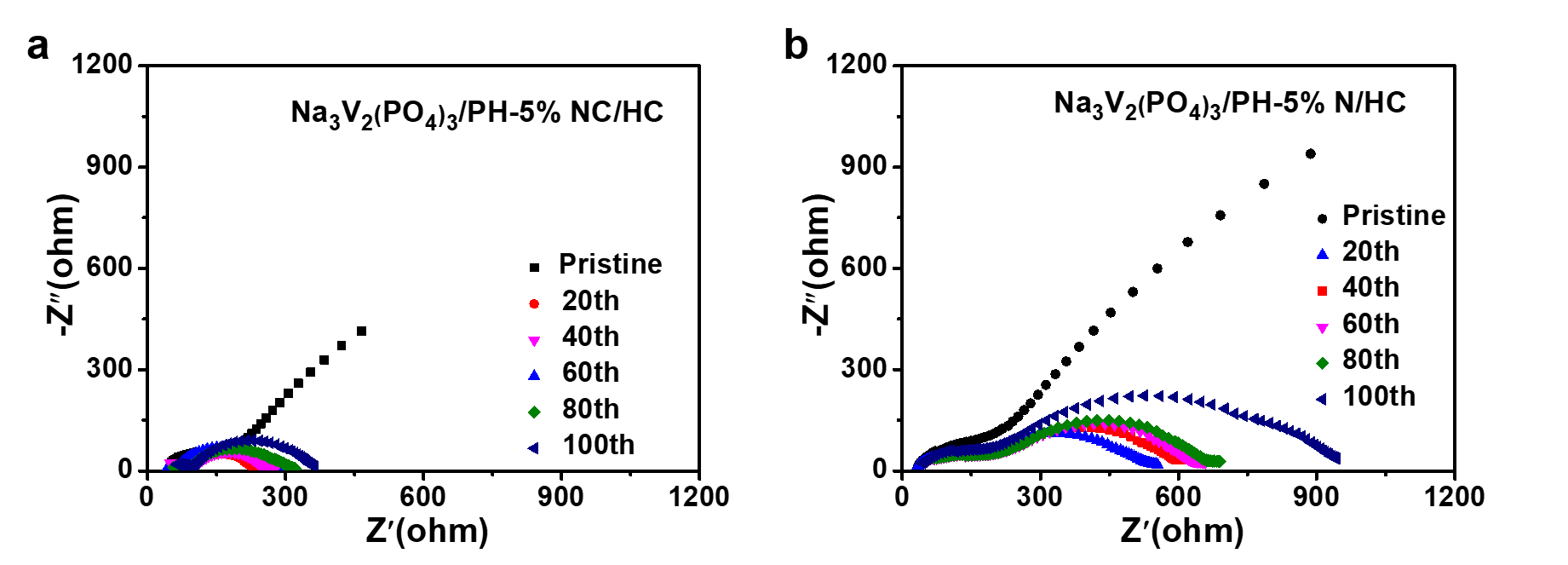


**Figure S24.** The EIS spectra of Na_3_V_2_(PO_4_)_3_//HC cells after different cycles at 1C under 25 °C.

[1] Y. Ebina, K. Akatsuka, K. Fukuda, T. Sasaki, *Chem. Mater.* **2012**, *24*, 4201.

[2] M. R. Aziza, C.-W. Chang, A. Mohapatra, C.-W. Chu, C.-C. Kaun, Y.-H. Su, *ACS Appl. Nano Mater.* **2020**, *3*, 6367.

[3] G. Rupprecht, R. Bell, *Phys. Rev. E* **1964**, *135*, A748.

[4] P. Song, T. Yang, Y. Ji, Z. Wang, Z. Yang, L. Chen, L. Chen, *Commun. Comput. Phys.* **2017**, *21*, 1325.

[5] P. Hohenberg, W. Kohn, *Phys. Rev* **1964**, 136, B864.

[6] W. Kohn, L. J. Sham, *Physical review* **1965**, 140, A1133.

[7] P. E. Blochl, *Physical Review-Section B-Condensed Matter* **1994**, 50, 17953.

[8] G. Kresse, J. Furthmüller, *Physical review B* **1996**, 54, 11169.

[9] J. P. Perdew, K. Burke, M. Ernzerhof, *Physical review letters* **1996**, 77, 3865.

[10] G. Henkelman, B. P. Uberuaga, H. Jónsson, *The Journal of chemical physics* **2000**, 113, 9901.

[11] J. Neugebauer, M. Scheffler, *Surface science* **1993**, 287, 572.
